# The Contribution of Noncommunicable and Infectious Diseases to the Effect of Depression on Mortality: A Longitudinal Causal Mediation Analysis

**DOI:** 10.1097/EDE.0000000000001804

**Published:** 2024-10-08

**Authors:** Christiane Didden, Matthias Egger, Naomi Folb, Gary Maartens, Eliane Rohner, Reshma Kassanjee, Cristina Mesa-Vieira, Ayesha Kriel, Soraya Seedat, Andreas D. Haas

**Affiliations:** From the a Institute of Social and Preventive Medicine, University of Bern, Bern, Switzerland; b Department of Sociology, LMU Munich, Munich, Germany; c Centre for Infectious Disease Epidemiology & Research, School of Public Health, University of Cape Town, Cape Town, South Africa; d Population Health Sciences, Bristol Medical School, University of Bristol, Bristol, UK; e Medscheme, Cape Town, South Africa; f Division of Clinical Pharmacology, Department of Medicine, University of Cape Town, Cape Town, South Africa; g Department of Psychiatry, Faculty of Medicine and Health Sciences, Stellenbosch University, Cape Town, South Africa; h South African Medical Research Council/Stellenbosch University Genomics of Brain Disorders Research Unit, Stellenbosch University, Cape Town, South Africa.

**Keywords:** Cancer, Cardiovascular diseases, Chronic respiratory diseases, HIV, Interventional effects, Major depressive disorder, Mediation, Tuberculosis

## Abstract

**Background:**

The increased prevalence of physical diseases among individuals with mental illness contributes to their increased risk of mortality. However, the mediating role of specific diseases in the effect of mental illness on mortality is not well understood.

**Method:**

We conducted a longitudinal causal mediation analysis using data from beneficiaries of a South African medical insurance scheme from 2011 to 2020. We estimated the overall effect of major depressive disorder (MDD) on mortality and evaluated reductions in this overall effect through hypothetical interventions on the risks of mediating physical diseases using an interventional effects approach. Monte Carlo simulation-based g-computation was used for estimation.

**Results:**

Among 981,540 individuals, 143,314 (14.6%) were diagnosed with MDD. Mortality risk after 8 years was 6.5% under MDD, and 5.3% under no MDD (risk ratio 1.23, 95% CI = 1.19, 1.26). Overall, 43.4% of this disparity could be attributed to higher rates of physical comorbidities due to MDD. Cardiovascular diseases accounted for 17.8%, followed by chronic respiratory diseases (8.6%), cancers (7.5%), diabetes and chronic kidney disease (5.8%), tuberculosis (4.3%), and HIV (2.7%).

**Conclusion:**

Within the privately insured population of South Africa, MDD is associated with increased mortality. We found that noncommunicable diseases, rather than infectious diseases, are important mediators of the effect of MDD on mortality.

Depression is a leading contributor to the global burden of disease (GBD). According to the GBD study, depressive disorders account for 11% of the global disease burden and 18% of the burden in South Africa.^1^ These estimates fail to capture the true disease burden,^1,2^ as they do not account for mortality indirectly caused by depression through its role in suicide and physical illness.^3–5^

In high-income countries, individuals with mental illnesses, especially those with severe conditions, experience mortality rates more than double those of the general population, with life expectancy reduced by up to 10 years.^5,6^ Increased mortality among people with mental illness can be attributed to their higher incidence of physical diseases, reduced access to or engagement with health care, and increased rates of suicide and fatal accidents.^3–5^ Approximately two-thirds of deaths among individuals with mental illness are due to natural causes, less than 20% to unnatural causes, and the remainder result from unknown causes.^6^ A study of privately insured individuals in South Africa has shown that men with depression have a life expectancy reduced by 4.3 years and women by 2.5 years.^7^ About 85% of this excess mortality burden is attributable to natural causes of death, including physical diseases and old age, and 15% to unnatural causes such as suicide or accidental deaths.^7^

In high-income countries, detailed cause-of-death data allow researchers to break down excess mortality from mental disorders by specific causes, with preventable noncommunicable diseases (NCDs) such as cardiovascular, respiratory, and alcohol-related liver diseases causing most deaths.^3,8^ In low- and middle-income countries, including South Africa, detailed cause-of-death data are typically not routinely collected, precluding such analyses. There is scarce evidence regarding the causes of excess mortality among individuals with mental illness in low- and middle-income countries, mostly derived from prospective population-based studies. One study from a rural Ethiopian region with high HIV and tuberculosis prevalence suggests infectious diseases are the main causes of excess mortality among those with severe mental illness.^9^ The specific causes mediating the effect of mental disorders on mortality in South Africa have not been studied and remain largely unknown,^7,10^ which hampers the development of effective interventions and policies to address this public health problem.

In this study, we aim to quantify the contribution of six leading causes of death in South Africa to the effect of major depressive disorder (MDD) on mortality. Leveraging longitudinal data from nearly one million South African medical insurance beneficiaries, we first conducted a causal analysis to investigate the overall effect of MDD on mortality using an interventional effects approach. We then conducted a causal mediation analysis to quantify the reductions in this overall effect achieved by setting the risks of the six physical diseases, assumed to mediate the effect of MDD on mortality, to the levels expected in the absence of MDD. The results of this study aim to determine which of these diseases should be prioritized for enhanced management or prevention strategies to reduce the increased mortality due to MDD.

## METHODS

### Study Design

**FIGURE 1.**
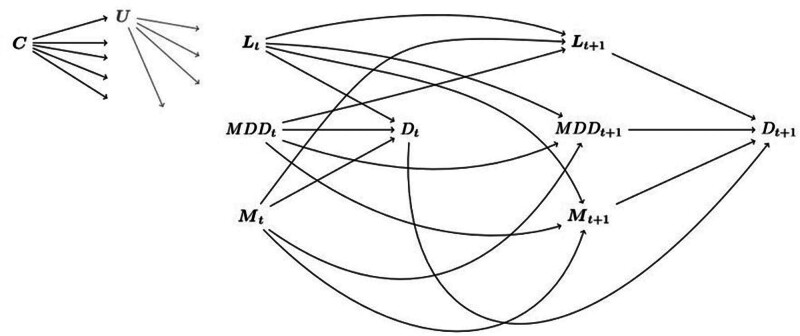
This directed acyclic graph (DAG) illustrates the assumed causal model of the effect of major depressive disorder (MDD) on mortality over two time points. For simplicity, earlier time points are not shown. Each node represents a variable or set of variables, and each arrow represents a causal effect with its direction. The diagram distinguishes between baseline covariates *C* (sex, age at enrollment, population group), the exposure MDD, mediators *M* (HIV, tuberculosis, diabetes and chronic kidney disease, cardiovascular diseases, chronic respiratory diseases, cancers), time-dependent confounders *L* (hypertension and anxiety disorders) and the outcome death (D). Unmeasured factors are represented by *U*. We operated under the following working assumptions: the presence of MDD at time period *t* can cause other medical conditions in the subsequent period *t+1*. At a given time period *t*, the medical conditions in *L*_*t*_, and *M*_*t*_ were treated as comorbidities of MDD_*t*_. We adjusted for these comorbidities when estimating the effect of MDD_*t*_ on D_*t*_ at *t* and the effect of MDD_*t*_ on other medical conditions at *t+1*. All conditions were treated as chronic, implying that a person diagnosed with a disease at time period *t* is considered to have the disease in later periods. Covariates in *C* and *U* may influence *L*, *M*, MDD, and D at each time interval.

We conducted a causal mediation analysis using longitudinal data from a cohort of South African medical insurance beneficiaries linked with vital registration data from South Africa’s National Population Register. The analysis was based on a directed acyclic graph (Figure 1) representing the presumed causal relationships between MDD, potential confounders, mediators, and mortality. Insurance data included outpatient, hospital, and pharmacy claims, and laboratory results. Diagnoses extracted from claims made in outpatient and hospital settings were coded according to the International Classification of Diseases, tenth revision (ICD-10). The Human Research Ethics Committee of the University of Cape Town and the Cantonal Ethics Committee of the Canton of Bern approved the study.

### Setting and Participants

We included beneficiaries of a private medical insurance scheme aged 18 or older who were covered by the scheme between 1 January 2011 and 15 March 2020. The insurance scheme offers managed care services, including disease and case management programs, and manages administrative functions, including claims processing, member enrollment, billing, and member communication. The scheme collaborates with many healthcare providers, including hospitals, doctors, and other healthcare facilities, serving almost four million South African residents. We excluded individuals with unknown age or sex and those who could not be linked to the National Population Register (eFigure 1, eAppendix; http://links.lww.com/EDE/C193).

### Procedures

The primary outcome was all-cause mortality, determined using mortality records from the National Population Register and the insurance database. In cases of discrepancies, we used the death dates from the National Population Register. Our exposure variable was defined by ICD-10 diagnoses of major depressive disorder (ICD-10 codes F32, F33). We considered six leading causes of death in South Africa:^11^ (1) HIV, (2) tuberculosis, (3) diabetes and chronic kidney disease, (4) cardiovascular diseases (CVDs), (5) chronic respiratory diseases, and (6) cancers. These causes were assessed as potential mediators of the effect of MDD on mortality. We considered neurotic, stress-related and somatoform disorders (ICD-10 codes F40–F48), which we referred to as anxiety disorders, and hypertension as potential time-dependent confounders (Figure 1). The case definitions for the physical conditions were based on ICD-10 codes from the GBD cause list,^12^ along with other indicators such as laboratory test results and medication claims indicating the presence of a disease. Further details on the case definitions can be found in the eAppendix; http://links.lww.com/EDE/C193, section 2. All medical conditions, including the primary exposure (MDD), were treated as time-dependent covariates. Individuals were considered “exposed” to a condition from the date of their first diagnosis onwards. Measured sociodemographic baseline characteristics, which may act as confounders or proxies for unmeasured confounders, include age at enrollment in the study, sex, and self-identified population group categorized as Black African, Indian/Asian, Mixed Ancestry, White, or unknown. Confounders and mediators were identified using the directed acyclic graph in Figure 1.

### Statistical Analysis

For each beneficiary, we divided time into 6-month intervals, up to a maximum follow-up duration of 8 years. Our model posits that the presence of a disease in any given interval *t* could have affected the risk of being diagnosed with another disease in the subsequent interval *t* + 1 (Figure 1).

We conducted two sets of analyses within a counterfactual framework using an interventional effects approach.^13–15^ All effects were defined by contrasts between the expected mortality of different counterfactual scenarios. Formal statistical definitions of the target parameters are provided in sections 4.2 and 4.3 of the eAppendix; http://links.lww.com/EDE/C193. In these scenarios, once an individual dies in a given period *t*, the individual remains deceased in all subsequent periods. We simulated the scenarios under no censoring; that is, at each time *t*, we set the censoring indicator, which indicates whether a person left the insurance for reasons other than death, to uncensored for each individual. We assumed noninformative censoring, as well as positivity and consistency.

First, we estimated an interventional overall effect of MDD on mortality. This effect encompasses direct and indirect pathways through which the exposure affects the outcome. We defined the overall effect as the difference in the expected mortality risk between two counterfactual scenarios: (A) The “always MDD” scenario (MDD = 1), in which each individual in the study population was exposed to MDD throughout the entire follow-up period. In this scenario, the values of the mediators and time-dependent confounders were set to random draws from their distributions in the study population under MDD = 1, given past time points and baseline covariates. (B) The “never MDD” scenario (MDD = 0), in which each individual was unexposed to MDD throughout the entire follow-up period. In this scenario, the values of the mediators and time-dependent confounders were set to random draws from their distributions in the study population under MDD = 0, given past time points and baseline covariates. The overall effect is presented as a difference in mortality percentage points and as a mortality risk ratio across various years of follow-up.

Second, we decomposed the overall effect into interventional indirect effects via the six causes of death. Interventional indirect effects are defined by (hypothetical) interventions on mediator distributions.^15–19^ For longitudinal survival settings with time-dependent confounding, indirect effects based on stochastic interventions on the mediators have been proposed by Zheng and van der Laan^19^ and Lin et al.,^13^ among others. Building on these methodologies, we estimated two types of interventional indirect effects: (1) an overall indirect effect through the six mediators considered jointly, and (2) indirect effects through single mediators. We defined the overall indirect effect as the contrast between the expected mortality risk in two scenarios: (A) the “always MDD” scenario, and (C) a scenario under always MDD where, at each time *t* from the second period onwards, the conditional distributions of the mediators were set equal to those that would have been observed if everyone had been un exposed to MDD. Specifically, scenario C was defined by setting each individual to MDD = 1 and assigning mediator values at *t =* 2, …,16 that were randomly drawn from the conditional mediator distributions at *t* under MDD = 0, given survival in period *t - 1* in scenario C. The distributions of the time-dependent confounders match those under always MDD, given past time points and baseline covariates. Thus, the shift in the distributions of the mediators at time *t* flow into the distributions of the time-dependent confounders at *t + 1*.

The indirect effect through a single mediator was defined as the contrast in the expected mortality risk between: (A) the “always MDD” scenario, and (D) a scenario under “always MDD,” where, at *t =* 2, …, 16, the conditional distribution of that single mediator, given survival in period *t - 1* in scenario D, was aligned with what would have been observed if everyone had been unexposed to MDD. This distributional shift at time *t* flow into the distributions of the time-dependent confounders and the other mediators at *t + 1*. This single-mediator analysis allowed us to evaluate which of the mediators’ distributional shifts would yield the most substantial reduction in the overall effect, providing valuable insights for potential prioritization.^18^

All indirect effects are given as percentages of the overall effect, interpreted as percentage reductions in the overall effect that could be achieved by the mediator interventions in scenarios C and D. In a sensitivity analysis based on a random 50% sample of our data, we relaxed the assumption that MDD at time *t* could only have affected the risk of being diagnosed with another disease in the subsequent period, instead assuming it might also have affected this risk at time *t*.

We used Monte Carlo simulation-based parametric g-computation for estimation (details in eAppendix; http://links.lww.com/EDE/C193, section 4.4).^15^ To assess the mediator distributions and the outcome distribution for the different time intervals, we fitted logistic regression models. In the model-building process for the outcome model for time *t* (*t =* 1,..., 16), we considered all diseases measured at *t* and all baseline characteristics. For the mediator models for time *t*, we considered all comorbidities measured in the previous time period *t - 1* and all baseline characteristics. Furthermore, we considered all possible two-way interaction terms. We utilized stepwise forward-backward selection with Akaike’s model selection criterion for model selection. We repeated the algorithm four times and averaged the results over the different Monte Carlo runs.^15^ Results are presented with 95% nonparametric bootstrap confidence intervals (CI). All analyses were stratified by sex and age group (<40 years, or ≥40 years). We chose the age cutoff at 40 years because this age typically signifies a higher risk of chronic illnesses and aligns with the recommendation to commence regular health checkups around the age of 40.^20–22^ Data management was done in Stata (Version 16. College Station, TX: StataCorp). Statistical analysis was done using R version 4.1.0 (R Foundation for Statistical Computing, Vienna, Austria). Calculations were performed on UBELIX (http://www.id.unibe.ch/hpc), the high-performance computing cluster at the University of Bern.

## RESULTS

We followed 981,540 individuals for a median duration of 3.1 years (interquartile range 1.2–6.2), resulting in a total of 3,727,993 person-years. During follow-up, 143,314 beneficiaries (14.6%) were diagnosed with MDD, and 34,230 (3.5%) died. Most deaths (87.0%) were due to natural causes. The characteristics of beneficiaries, stratified by whether they received an MDD diagnosis during follow-up, are shown in the Table. The mean age at the start of follow-up was 39.3 years (standard deviation 15.3), and 51.7% were female. At the end of follow-up, 8.4% had been diagnosed with HIV, 1.9% with tuberculosis, 10.4% with CVDs, 4.0% with cancers, 11.4% with diabetes, 3.3% with chronic kidney disease, 9.5% with chronic respiratory diseases, 30% with hypertension, and 17% with anxiety disorders. The number of participants under follow-up at the end of each year stratified by whether an MDD diagnosis was received by the end of the year or before, is shown in eTable 1 in the eAppendix; http://links.lww.com/EDE/C193.

**TABLE.** Characteristics of Beneficiaries, Stratified by Whether They Received a Major Depressive Disorder Diagnosis During Follow-up

	Total	Major Depressive Disorder Diagnosis	No Major Depressive Disorder Diagnosis
	N = 981,540 (100.0)	N = 143,314 (14.6)	N = 838,226 (85.4)
Characteristics at baseline			
Age, years			
18–39	545,712 (55.6)	70,780 (49.4)	474,932 (56.7)
40+	435,828 (44.4)	72,534 (50.6)	363,294 (43.3)
Mean (SD)	39.3 (15.3)	41.4 (15.2)	38.9 (15.2)
Sex			
Male	474,516 (48.3)	51,671 (36.1)	422,845 (50.4)
Female	507,024 (51.7)	91,643 (63.9)	415,381 (49.6)
Population group			
Black African	507,672 (51.7)	61,342 (42.8)	446,330 (53.2)
Mixed Ancestry	63,268 (6.4)	9393 (6.6)	53,875 (6.4)
White	177,817 (18.1)	41,125 (28.7)	136,692 (16.3)
Indian/Asian	44,217 (4.5)	5236 (3.7)	38,981 (4.7)
Unknown	188,566 (19.2)	26,218 (18.3)	162,348 (19.4)
Characteristics at the end of follow-up			
Physical diseases			
HIV	82,636 (8.4)	16,756 (11.7)	65,880 (7.9)
Tuberculosis	19,052 (1.9)	4464 (3.1)	14,588(1.7)
CVDs	102,316 (10.4)	29,908 (20.9)	72,407 (8.6)
Cancers	38,810 (4.0)	9693 (6.8)	29,116 (3.5)
Diabetes	111,453 (11.4)	26,550 (18.5)	84,903 (10.1)
Chronic kidney disease	32,375 (3.3)	8323 (5.8)	24,051 (2.9)
Chronic respiratory diseases	92,957 (9.5)	26,452 (18.5)	66,505 (7.9)
Hypertension	294,371 (30.0)	69,058 (48.2)	225,313 (26.9)
Psychiatric comorbidities			
Organic mental disorder	9101 (0.9)	4762 (3.3)	4339 (0.5)
Substance use disorder	9715 (1.0)	5234 (3.7)	4481 (0.5)
Serious mental disorder	20,851 (2.1)	15,800 (11.0)	5051 (0.6)
Other mood disorders	4946 (0.5)	3076 (2.1)	1870 (0.2)
Anxiety disorders	169,107 (17.2)	70,905 (49.5)	98,202 (11.7)
Other mental disorders	21,96 (2.2)	9243 (6.4)	12,717 (1.5)
Mortality	34,230 (3.5)	6550 (4.6)	27,680 (3.3)
Natural causes	29,767 (3.0)	5791 (4.0)	23,975 (2.9)
Unnatural causes	3043 (0.3)	530 (0.4)	2511 (0.3)
Unknown	1427 (0.1)	229 (0.2)	1194 (0.1)
Follow-up time, years			
Median (IRQ)	3.1 (1.2–6.2)	5.5 (3.1–9.0)	2.7 (1.1–5.4)

Data are n (%) unless otherwise stated.

The denominator for percentages within each group is the total number of individuals in that group.

CVDs indicates cardiovascular diseases; IQR, interquartile range; SD, standard deviation.

**FIGURE 2.**
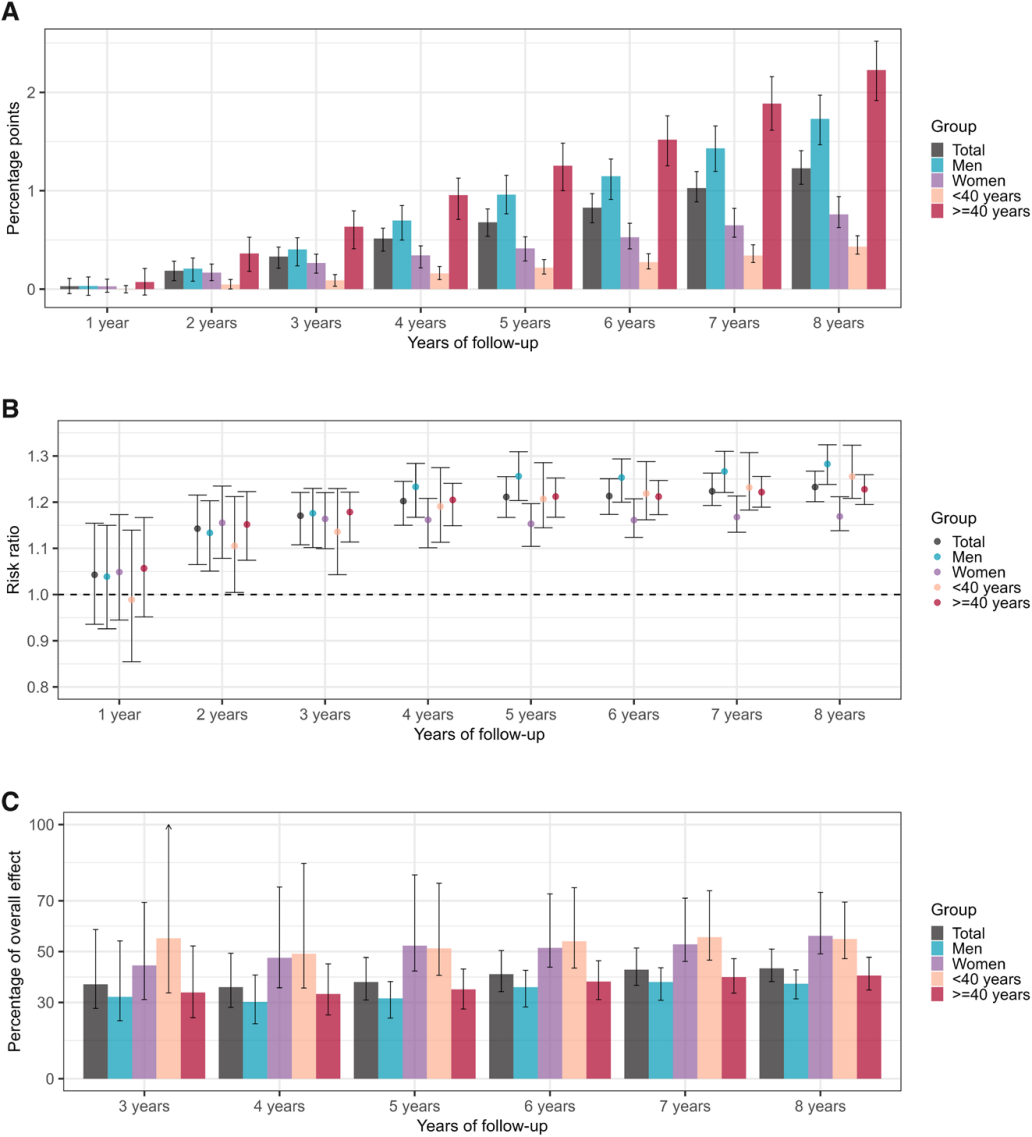
Interventional overall effect of major depressive disorder (MDD) on mortality and the interventional overall indirect effect. (A and B) show the estimated interventional overall effects at 1–8 years of follow-up defined as the difference in all-cause mortality risk between two counterfactual scenarios: everyone being exposed to MDD (“always MDD” scenario) versus no-one being exposed to MDD throughout follow-up (“never MDD” scenario). (A) An overall effect as an absolute difference in mortality as percentage points, while (B) the relative difference in mortality expressed as risk ratio. (C) The interventional overall indirect effects at 3–8 years of follow-up defined as the percentage reductions in the interventional overall effect achieved by setting the risks of HIV, tuberculosis, diabetes, chronic kidney disease, cardiovascular diseases (CVDs), chronic respiratory diseases, and cancers under always MDD equal to those that would be observed in the absence of MDD, i.e., in the “never MDD” scenario. The error bars indicate the 95% confidence intervals.

**FIGURE 3.**
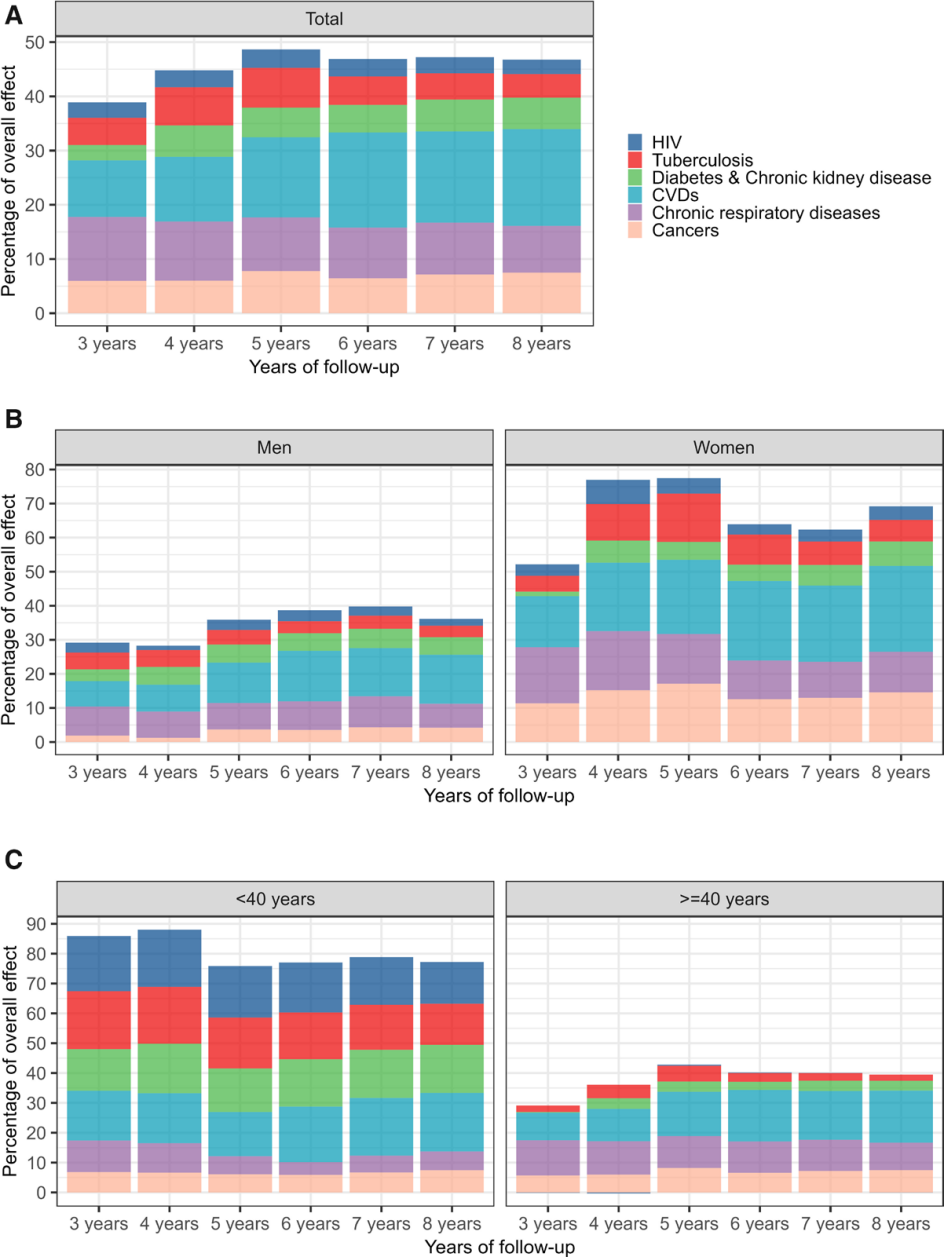
This figure shows the estimated interventional indirect effects of major depressive disorder (MDD) on mortality through six physical diseases at 3-8 years of follow-up. The interventional indirect effect through a physical disease in a given year is defined as the reduction in the mortality risk under always MDD for that year, achieved by setting the risk of that disease over the entire follow-up period to the level that would be observed in the absence of MDD, i.e., in the “Never MDD” scenario. The colored bars represent the indirect effects as percentages of the interventional overall effect of MDD on mortality. (A) Results for the entire study population, (B) stratified by sex, and (C) by age groups.

**FIGURE 4.**
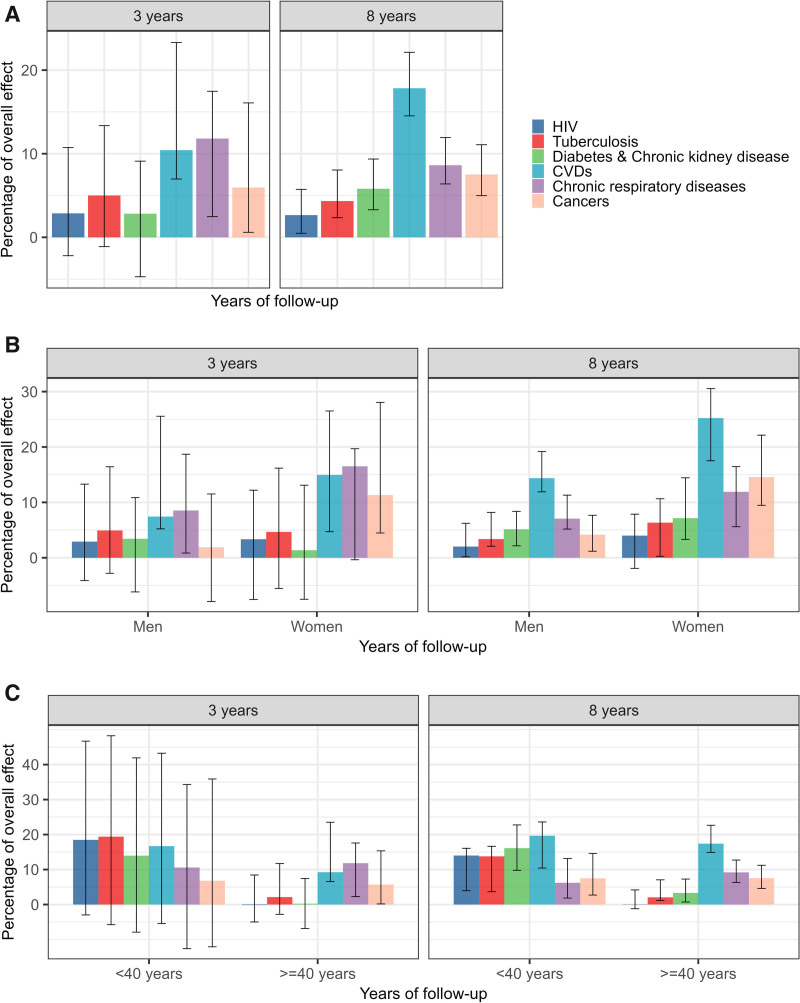
This figure shows the estimated interventional indirect effects of major depressive disorder (MDD) on mortality through six physical diseases at year 3 and year 8 of follow-up with 95% confidence intervals. This figure shows the estimated interventional indirect effects of MDD on mortality through six physical diseases at year 3 and year 8 of follow-up with 95% confidence intervals. The interventional indirect effect through a physical disease in a given year is defined as the reduction in the mortality risk under always MDD for that year, achieved by setting the risk of that disease over the entire follow-up period to the level that would be observed in the absence of MDD. The colored bars represent the indirect effects as percentages of the interventional overall effect of MDD on mortality. (A) Results for the entire study population, (B) stratified by sex, and (C) by age groups.

In the “always MDD” scenario, 63,846 individuals (6.5%, 95% CI = 6.3, 6.7) had died after 8 years. In contrast, in the “never MDD” scenario, 51,791 individuals (5.3%, 95% CI = 5.2, 5.4) had died over the same period. The mortality risk ratio comparing the two scenarios increased from 1.14 (95% CI = 1.06, 1.22) at 2 years to 1.23 (95% CI = 1.20, 1.27) at 8 years (Figure 2B). After 8 years of follow-up, the estimated overall indirect effect of MDD on mortality through the six physical comorbidities accounted for 43.4% (95% CI = 38.2, 51.0) of the overall effect (Figure 2C). From the fourth year onwards, the indirect effects through CVDs were the largest (Figure 3A). At 8 years, the overall effect of MDD on mortality would have reduced by 17.8% (95% CI = 14.5, 22.1) if, throughout follow-up, the conditional distribution of CVDs under MDD had been set to the level that would have been observed in the absence of MDD. The second largest indirect effect was the indirect effect through chronic respiratory diseases (8.6%, 95% CI = 6.4, 11.9), followed by those through cancers (7.5%, 95% CI = 5.0, 11.1), diabetes and chronic kidney disease (5.8%, 95% CI = 3.3, 9.4), tuberculosis (4.3%, 95% CI = 2.3, 8.1), and HIV (2.7%, 95% CI = 0.5, 5.7) (Figure 4A).

The estimated effect of MDD on mortality was higher among men than among women. After 8 years, in the “always MDD” scenario, 37,249 of the men (7.8%, 95% CI = 7.6, 8.1) and 26,597 of the women (5.2%, 95% CI = 5.1, 5.4) had died. In the “never MDD” scenario, 29,042 of the men (6.1%, 95% CI = 6.0, 6.2), and 22,749 of the women (4.5%, 95% CI = 4.4, 4.6) had died over the same period. The mortality risk ratio after 8 years was 1.28 (95% CI = 1.24, 1.32) in men and 1.17 (95% CI = 1.14, 1.21) in women (Figure 2B). The estimated overall indirect effect accounted for a higher proportion of the overall effect in women (56.2%, 95% CI = 49.1, 73.3, at 8 years) than in men (37.3%, 95% CI = 31.4, 42.8, at 8 years) (Figure 2C). The largest indirect effect was that through CVDs in both men and women. The indirect effect through cancers was the second largest among women, while its impact was relatively small among men. Among men, the indirect effect through chronic respiratory diseases ranked second (Figures 3B and 4B).

After 8 years, in the “always MDD” scenario, 11,569 individuals in the <40 age group (2.1%, 95% CI = 2.0, 2.3) and 52,277 individuals in the ≥40 age group (12.0%, 95% CI = 11.7, 12.3) had died. In the “never MDD” scenario, 9214 individuals in the <40 age group (1.7%, 95% CI = 1.6, 1.7), and 42,577 individuals in the ≥40 age group (9.8%, 95% CI = 9.6, 9.9) had died. The risk ratio was 1.26 (95% CI = 1.21, 1.32) in the younger adults and 1.23 (95% CI = 1.19, 1.26) in the older adults (Figure 2B). In individuals <40 years, the overall indirect effect through the mediators accounted for a larger proportion of the overall effect (54.9%, 95% CI = 47.2, 69.5) at 8 years than in individuals ≥40 years (40.6%, 95% CI = 34.9, 47.8) (Figure 2C). In the younger age group, the indirect effects via CVDs, diabetes, and chronic kidney disease were largest at 8 years, followed by those via tuberculosis and HIV. Among older adults, the indirect effects through infectious diseases were relatively small, and those through CVDs, chronic respiratory diseases, and cancers were the largest (Figures 3C and 4C).

In the sensitivity analysis, based on the assumption that MDD at time *t* might affect the risk of being diagnosed with another disease at *t*, the overall effects and the overall indirect effects of MDD on mortality were generally larger than in the main analysis. The single-mediator analysis reinforces the finding that NCDs, especially CVDs, were important mediators, while also highlighting the relevance of infectious diseases in the younger age group (eFigures 2–4, eAppendix; http://links.lww.com/EDE/C193).

## DISCUSSION

This study documents a substantially increased mortality risk due to MDD among privately insured individuals in South Africa, with more pronounced increases in men compared with women. The elevated risks of the six modeled physical diseases under MDD contributed substantially to the estimated overall effect of MDD on mortality, mediating more than 43% of this effect. In particular, NCDs, especially CVDs, were important mediators. Infectious diseases were relevant mediators in adults under 40 years of age but played a minor role overall. The remaining 57% of the estimated overall effect of MDD on mortality, not attributed to the elevated risk of the six diseases under MDD, was likely due to other causes of death not modeled in this study, including unnatural causes such as accidents, suicide, and homicide as well as other physical diseases, such as pneumonia and influenza. These causes account for 56% of the deaths in South Africa in 2018, whereas the six modeled causes accounted for 44%.^11^

Several biologic and behavioral mechanisms support the plausibility of our results. First, depression is associated with hormonal abnormalities, which can result in compromised immune functioning.^23–27^ Second, depression may lead to risky behaviors such as unsafe sex, substance use, poor diet, and low physical activity, which increase the risk of physical illness.^28–30^ Third, depression may affect individuals’ care-seeking behavior and adherence to chronic medications.^31^ Finally, depression can lead to social isolation and a lack of social support,^32^ which has a negative impact on physical well-being.^33,34^

The observed differences in the indirect effects of MDD on mortality in men and women might be influenced by biologic factors, as well as behavioral, social, and structural factors shaped by gender roles. Further investigation is needed to understand the specific mechanisms by which MDD impacts men and women differently, which is crucial for developing gender-sensitive interventions aimed at reducing excess mortality due to MDD.

Our findings align with previous high-income country data emphasizing higher mortality rates in individuals with depression. A Danish study with a similar age distribution to those in our data reported a mortality rate ratio of 1.92 in individuals with mood disorder such as depression compared with the general population. The study reported that men with mood disorders died 7.9 years earlier on average, and women 6.2 years earlier, compared with their counterparts in the general population. Furthermore, NCDs were identified as important causes of death contributing to excess mortality associated with mood disorders.^8^ The mortality among individuals with MDD in our cohort was substantially lower than the mortality among individuals with mood disorders in the Danish study. This disparity could stem from including a broader spectrum of individuals with MDD in our study, which comprises 15% of our study population, including those with milder depression diagnosed in outpatient settings. The Danish study reported a 3.1% mood disorder prevalence. Unlike our study, they did not include patients with milder disorders treated solely by general practitioners. Our findings differ from evidence from rural Ethiopia, showing high mortality in individuals with severe mental disorders, including severe depression, mainly due to infectious diseases, accounting for 50% of deaths.^9^ High mortality from infectious diseases likely reflects a high HIV prevalence and limited HIV treatment coverage during the study period. In our South African private sector cohort, characterized by high HIV treatment coverage and high viral suppression rates,^31^ HIV and tuberculosis were not major mediators of the estimated effect of MDD on mortality.

When interpreting our study results, certain limitations must be considered. First, our study relied primarily on ICD-10 diagnoses from claims data from a medical insurance scheme to identify medical conditions. While such administrative data can be prone to inaccuracies and misclassification, it has been shown that depression diagnoses from administrative sources generally have a high positive predictive value compared with research diagnoses.^35^ Second, by identifying a medical condition based on ICD-10 diagnoses and procedure codes, we missed undiagnosed cases and those who died before receiving medical attention. If only more severe MDD cases are diagnosed and mild cases remain undiagnosed, this might lead to an overestimation of the effect of MDD on mortality. However, our data include diagnoses from all levels of care, including primary care, and show a high prevalence of depression and physical disorders modeled as mediators. This suggests that substantial underdiagnosis of these conditions is unlikely. Third, using diagnosis dates as proxies for disease onset could distort the temporal order of disease occurrence, which is critical for causal inference. For example, if MDD tends to be diagnosed late compared with other diseases, we may have underestimated the impact of MDD on mortality and other diseases. Another issue concerning the temporal order of diagnoses is our conservative approach to the causal effect of MDD on other diseases. We assumed that MDD diagnosed within a specific period *t* could only influence diseases diagnosed in subsequent periods. Our sensitivity analysis indicates that the estimated effects of MDD on mortality would be larger if we had allowed for the possibility that MDD diagnosed at time *t* may have affected other diseases diagnosed during the same time period. Fourth, beneficiaries who left the scheme before the end of the follow-up were right-censored, assuming that censoring was noninformative. This assumption may be violated if, for example, the decision to leave or remain in the scheme is influenced by the presence of any of the conditions under consideration or other causes of mortality. For example, if individuals lose their employment and, as a consequence, their insurance coverage due to severe depression, this could result in an underestimation of the effect of MDD on mortality, particularly if individuals with more severe depression have a higher risk of mortality than those with less severe depression.

Regarding the causal interpretation of the estimates, it is important to acknowledge that a causal interpretation of the estimated overall effect of MDD on mortality is only valid if there are no unmeasured confounders affecting both MDD and mortality. In addition, for the estimated indirect effects through physical illness to have causal meaning, there must be no unmeasured confounders of the exposure-mediator and mediator-outcome relationships.^16^ Unmeasured confounding may be introduced by unmeasured medical conditions, especially those from the preinsurance period, health behaviors, as well as unmeasured parameters for the socio-economic status (SES) of the individuals, which are known to be associated with risk factors for depression,^36,37^ physical conditions, and mortality.^38,39^ For example, if low SES increases the risk of MDD, physical illness, and death, the effect of MDD on mortality would be overestimated without adjustment for SES. In our study of privately insured individuals in South Africa, the variance in SES-related factors is likely to be relatively small compared to the general population of South Africa. Most of our study participants likely belong to the two highest wealth quintiles, and there is no evidence of differences in the prevalence of mental disorders between these two quintiles.^40^ Therefore, we assume that unaccounted SES differences should not substantially bias our results. However, our findings should generally be interpreted cautiously, as we cannot exclude the possibility of bias from unmeasured confounding. We must also acknowledge potential violations of the positivity assumptions, under which we assumed a positive probability of receiving a disease diagnosis across all relevant covariate strata. We tried to reduce the impact of potential violations of the positivity assumptions through flexible modeling. The use of doubly robust methods and machine learning algorithms could further minimize the risk of model misspecification.^41^ However, the development and application of these methods in complex longitudinal mediation analyses are still ongoing. Additionally, there have been concerns raised regarding the interpretation of interventional indirect effects in a strict mediational sense. It has been argued that such interpretations require further strong assumptions,^42^ which may not be satisfied in the context of our study. Nevertheless, we propose that the indirect effects estimated in this study capture mediational concepts, as they quantify the proportion of the overall effect of MDD on mortality attributable to the elevated risk of physical comorbidity under MDD.^16^

In conclusion, our study is consistent with the hypothesis that MDD increases mortality within the privately insured population of South Africa. Despite the high HIV and tuberculosis prevalence in the region, our findings estimate that these infectious diseases do not substantially mediate the effect of MDD on mortality; instead, NCDs, particularly CVDs, are important mediators.

## ACKNOWLEDGMENTS

Calculations were performed on UBELIX (http://www.id.unibe.ch/hpc), the high-performance computing cluster at the University of Bern.

During the preparation of this work, the authors used ChatGPT-4 and Grammarly to correct grammar and spelling and to improve the clarity and flow of the writing. After using this tool, the authors reviewed and made necessary edits, taking full responsibility for the content of the publication.

## Supplementary Material
