# Supplementary material for: The Contribution of Noncommunicable and Infectious Diseases to the Effect of Depression on Mortality: A Longitudinal Causal Mediation Analysis
Source: Epidemiology. 2024 Oct 8;36(1):88–98. doi: 10.1097/EDE.0000000000001804 (PMC11594557; doi:10.1097/EDE.0000000000001804)
Supplement: Supplementary file 1 [file ede-36-88-s001.pdf]

The contribution of non-communicable and infectious diseases to the effect of depression on mortality: a longitudinal causal mediation analysis

eAppendix

1 Flow diagram of inclusion of individuals in study

eFigure 1

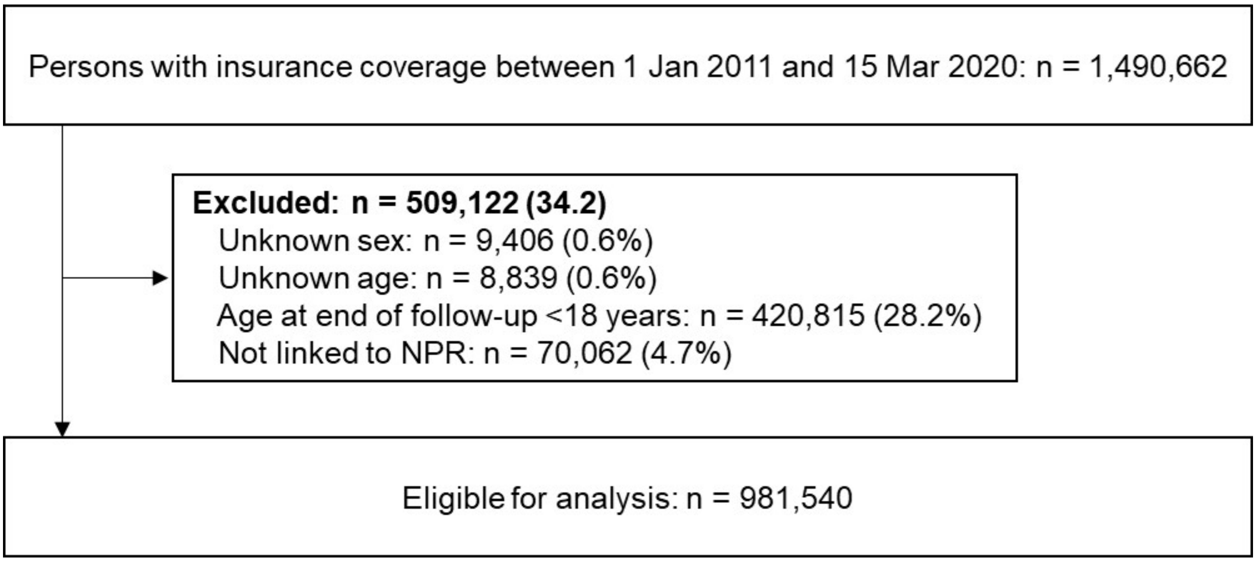

2 Case definitions

HIV was defined based on laboratory data for HIV viral load, CD4 count, or a positive confirmatory HIV test, an ICD-10 diagnosis for HIV or the use of antiretroviral medication for treating HIV excluding medication commonly used in pre- or post-exposure prophylaxis (Table S1).

Table S1: List of diagnoses, medications, and test results indicative of HIV

Diagnoses	ICD10 code
Human immunodeficiency virus (HIV) disease	B20-B24
Asymptomatic HIV infection status	Z21
Laboratory evidence of HIV	R75
HIV disease complicating pregnancy, childbirth, and the puerperium	O98.7
**Antiretroviral medication for treating HIV**	**ATC code**
Protease inhibitors	J05AE
Nucleoside and nucleotide reverse transcriptase inhibitors	J05AF
Non-nucleoside reverse transcriptase inhibitors	J05AG
Integrase inhibitors	J05AJ
Antivirals for treatment of HIV infections, combinations	J05AR
**Antiretroviral medication used in pre- or post-exposure prophylaxis**	**ATC code**
Tenofovir disoproxil and emtricitabine (TDF/FTC)	J05AR03
Tenofovir alafenamide (TAF)	J05AF13
Emtricitabine (FTC)	J05AF09
Lamivudine (3TC)	J05AF05
**Laboratory test**	**Value**
Confirmatory HIV test	Positive

Tuberculosis was defined based on ICD-10 codes for tuberculosis, evidence of use of medication used to treating tuberculosis (ATC codes J04A), or a positive tuberculosis PCR test (Table S2).

Table S2: List of diagnoses, medications, and test results indicative of Tuberculosis

Diagnoses	ICD10 code
Tuberculosis	A15-A19
Sequelae of tuberculosis	B90
Tuberculous oesophagitis	K23.0
Tuberculous peritonitis	K67.3
Tuberculous disorders of intestines, peritoneum and mesenteric glands	K93.0
Tuberculous arthritis	M01.1
Tuberculosis of spine	M49.0
Tuberculosis of bone	M90.0
Tuberculous cystitis	N33.0
Tuberculous infection of cervix uteri	N74.0
Female tuberculous pelvic inflammatory disease	N74.1
Congenital tuberculosis	P37.0
Resistance to tuberculostatic drug(s)	U84.3
**Drug**	**ATC code**
Drugs for treatment of tuberculosis	J04A
**Laboratory results**	**Value**
Tuberculosis PCR	Positive

Cardiovascular diseases were defined based on ICD-10 diagnoses used in GBD's cased definition (Table S3).

Table S3: List of diagnoses of cardiovascular disease

Diagnoses	ICD10 code
Ischemic heart disease	I20-I25
Cerebrovascular disease (referred to as stroke in the GBD study)	G45-G46, I60-I62, I62.9-I69
Hypertensive heart disease	I11

Chronic respiratory diseases were defined based on ICD-10 diagnoses used in GBD's cased definition (Table S4).

Table S4: List of diagnoses of chronic respiratory disease

Diagnoses	ICD10 code
Chronic obstructive pulmonary disease	J41-J44
Asthma	J45-J46

Diabetes mellitus was defined based on ICD-10 codes for diabetes (E10-E14), evidence of use of medications used for diabetes control (ATC codes A10), or at least two abnormal laboratory results of HbA1c ≥6.5% (≥48 mmol/L), fasting blood glucose ≥7 mmol/L or random blood glucose ≥11.1 mmol/L (Table S5).

Table S5: List of diagnoses, medications, and laboratory test results indicative of diabetes mellitus

**Diagnoses**	**ICD10 code**
Type 1 diabetes mellitus	E10.0-1, E10.3-9
Type 2 diabetes mellitus	E11.0-1, E11.3-9
Malnutrition-related diabetes mellitus	E12.0-1, E12.3-9
Other specified diabetes mellitus	E13.0-1, E13.3-9
Unspecified diabetes mellitus	E14.0-1, E14.3-9
**Drug**	**ATC code**
Drugs used in diabetes	A10
**Laboratory test**	**Value**
HbA1c	≥6.5 % (≥48mmol/L)
Fasting blood glucose	≥7.0 mmol/L (~126 mg/dL)
Random blood glucose	≥11.1 mmol/L (~200 mg/dL)

Chronic kidney disease was defined based on ICD-10 codes and procedure codes for renal dialysis (Table S6).

Table S6: List of diagnoses of chronic kidney disease

**Diagnoses**	**Code type**	**Code**
Diabetes mellitus with renal complications	ICD-10	E10.2, E11.2, E12.2, E13.2, E14.2
Hypertensive renal disease	ICD-10	I12
Hypertensive heart and renal disease	ICD-10	I13
Glomerular diseases	ICD-10	N02-N08
Balkan nephropathy	ICD-10	N15.0
Renal failure	ICD-10	N17-N19
Congenital malformations of the urinary system	ICD-10	Q60-Q63.2, Q63.8-Q63.9, Q64.2-Q64.9
**Procedures**		
Care involving dialysis	ICD-10	Z49
Dependence on renal dialysis	ICD-10	Z99.2
Renal dialysis	NRPL	75146, 75148

Cancers were defined based on ICD-10 diagnoses used in GBD's cased definition (Table S7).

Table S7: List of diagnoses of cancer

**Diagnoses**	**Code type**	**Code**
Malignant neoplasm except other malignant neoplasms of skin (C44)	ICD-10	C00-C43, C45-C97

Hypertension was defined based on ICD-10 diagnoses, evidence of use of medications used for hypertension control (ATC codes), or at least two abnormal laboratory results of systolic blood pressure (≥140mmHg) and diastolic blood pressure (≥90mmHg) (Table S8).

Table S8: List of diagnoses of hypertension

**Diagnoses**	**ICD10 code**
Essential hypertension	I10
Hypertensive heart disease	I11
Hypertensive renal disease	I12
Hypertensive heart and renal disease	I13
Secondary hypertension	I15
Hypertensive retinopathy	H35.0
Hypertensive encephalopathy	I67.4
**Drug**	**ATC code**
Low-ceiling diuretics (thiazides)	C03A
Low-ceiling diuretics (non-thiazides)	C03B
Low-ceiling diuretics in combination with potassium-sparing agents	C03EA
Beta-blockers combined with thiazides	C07B
Beta-blockers with "other" diuretics	C07C
Beta-blockers with thiazide diuretic with "other" diuretics	C07D
Calcium channel blockers in combination with diuretics	C08G
ACE-inhibitors with diuretics	C09BA
Angiotensin II receptor-blockers with diuretics	C09DA
Valsartan + amlodipine + hydrochlorothiazide	C09DX01
Olmesartan + amlodipine + hydrochlorothiazide	C09DX03
Candesartan + amlodipine + hydrochlorothiazide	C09DX06
Aliskiren + hydrochlorothiazide	C09XA52
Aliskiren + amlodipine + hydrochlorothiazide	C09XA54
Rosuvastatin + perindopril + indapamide	C10BX13

Clinical test	Value
Systolic blood pressure	≥140mmHg
Diastolic blood pressure	≥90mmHg

Anxiety disorders were defined based on ICD-10 diagnoses for neurotic, stress-related and somatoform disorders (F40-F48).

3 Descriptive Statistics

eTable 1: Number of participants with follow-up at the end of each year, stratified by whether they received an MDD diagnosis at the end of the year or before.

Year	Total	MDD	No MDD
1	884,024	48,160	835,864
2	691,032	63,253	627,779
3	560,836	69,780	491,056
4	407,298	62,745	344,553
5	337,855	61,695	276,160
6	279,351	58,423	220,928
7	232,358	54,336	178,022
8	176,017	45,434	130,583

4 Causal analysis

4.1 Observed data

Let A represent the exposure, major depressive disorder (MDD), D the outcome, death, and $\boldsymbol{M}$ the set of indicators for six time-dependent mediators: HIV (M_1), tuberculosis (M_2), diabetes and chronic kidney disease (M_3), cardiovascular diseases (CVDs) (M_4), chronic respiratory diseases (M_5), and cancers (M_6). Let $\boldsymbol{L}$ denote the set of indicators for the time-dependent comorbidities anxiety disorders (L_1) and hypertension (L_2), which may confound the exposure-outcome, exposure-mediator, or mediator-outcome relationships. Since these comorbidities may themselves be affected by prior exposure, they may also act as mediators (see Figure 1, main paper). Let $\boldsymbol{W}$ denote the combined set of $\boldsymbol{M}$ and $\boldsymbol{L}$, i.e., $\boldsymbol{W} = (\boldsymbol{M}, \boldsymbol{L}) = (M_1, \dots, M_6, L_1, L_2) = (W_1, \dots, W_8)$. Further, let $\boldsymbol{C}$ represent the set of baseline covariates: sex (C_1), age at enrollment in the study (C_2), and population group (C_3). The indices assigned to the variables in $\boldsymbol{W}$ and $\boldsymbol{C}$ are arbitrary and do not imply any assumptions about the causal order of the variables. For any set of variables $\boldsymbol{X} = (X_1, \dots, X_M)$, let $\boldsymbol{X}_{(-m)}$ denote the subset without the mth element. Further, let Z denote the indicator for censoring at the end of a time period, excluding censoring due to death.

There are 16 time periods in total, i.e., $T = 16$, with each time period spanning six months. Let $(A(1), ..., A(16))$, $(M_k(1), ..., M_k(16))$ for all $k \in \{1, \dots, 6\}$, $(L_j(1), ..., L_j(16))$ for all $j \in \{1, 2\}$, and $Z(1), \dots, Z(16)$ denote random variables corresponding to the time-dependent covariates in all measurement periods $t = 1, \dots, 16$, with initial baseline covariates $\boldsymbol{C}$. All medical conditions in A and $\boldsymbol{W}$ are treated as chronic throughout the follow-up period. For instance, when $A(t) = 1$ (i.e., MDD by time period t), $A(t+1), \dots, A(16)$ are all set to one. Similarly, when $D(t) = 1$ (i.e., death by time period t), $D(t+1), \dots, D(16)$ remain at one, and medical conditions from $t+1$ to 16 are undefined. We assume that a medical condition diagnosed in time period t may have affected other medical conditions diagnosed at $t+1$ (see Figure 1, main text). The medical conditions in the first time period are treated as baseline comorbidities.

4.2 Hypothetical interventions

4.2.1 Hypothetical static interventions on the exposure and on censoring

Let $\bar{A}(t) = \bar{a}(t)$ denote an intervention on the exposure, where we set $A(1), \dots, A(t)$ to $a(1), \dots, a(t)$. Specifically, we define the following two hypothetical interventions: "always exposed to MDD", where $\bar{A}(T) = \bar{a}(16) = \bar{1}(16) = \bar{1} = (1, \dots, 1)$, and "never exposed to MDD", where $\bar{A}(T) = \bar{a}(16) = \bar{0}(16) = \bar{0} = (0, \dots, 0)$. Additionally, we consider interventions on censoring, denoted by $\bar{Z}(t) = \bar{z}(t)$. Specifically, we set Z to "never censored", where $\bar{Z}(T) = \bar{z}(16) = \bar{0}$. We use superscripts to denote counterfactual variables and distributions. For any variable X, $X(t)^{\bar{z}=\bar{0},\bar{a}}$ denotes the counterfactual value of X, and $P(X(t)^{\bar{z}=\bar{0},\bar{a}})$ denotes the counterfactual distribution of X at time period t under a scenario of no censoring,

with exposure values set to $\bar{a}(t)$.

4.2.2 Hypothetical stochastic mediator interventions

As suggested for longitudinal mediation analyses by Lin et al. [1], VanderWeele and Tchetgen Tchetgen [2], Zheng and van der Laan [3] and others, we define hypothetical stochastic interventions on the mediators, characterized by setting their values to random draws from specific intervention distributions. For each time period, and similar to what has been proposed by Zheng and van der Laan [3], we specify mediator intervention distributions for the counterfactual scenarios under never MDD and under always MDD, conditional on baseline covariates, past counterfactual comorbidities and survival. Unlike in Zheng and van der Laan [3], however, our exposure (MDD) is time-dependent rather than time-fixed. In contrast to the causal models in Lin et al. [1] and VanderWeele and Tchetgen Tchetgen [2], we assume that the exposure at time period t can only affect the mediators at the subsequent time period $t+1$.

Adapting the notation used by Moreno-Betancur et al. [4] for stochastic mediator interventions to our longitudinal setting, we let $\bar{B}(t)$ denote a hypothetical stochastic intervention targeting the mediating conditions in $\boldsymbol{W} = (\boldsymbol{M}, \boldsymbol{L})$ up to and including time t. Furthermore, we let $\widetilde{W}_s(t)^{\bar{b}}$ denote a random draw for W_s $(s = 1, \ldots, 8)$ at time period t from a distribution specified by $\bar{B}(t)$, and $\widetilde{\boldsymbol{W}}(t)^{\bar{b}}$ represent the vector of random draws $\widetilde{W}_1(t)^{\bar{b}}, \ldots, \widetilde{W}_8(t)^{\bar{b}}$. The notation $D(t)^{\bar{z}=\bar{0},\bar{a},\bar{b}}$ refers to the counterfactual outcome (death) at time t in a counterfactual scenario without censoring, where the exposure is set to $\bar{a}(t)$ and $W_1, \ldots, W_8$ are set to random draws from the distributions specified by $\bar{B}(t)$.

We consider four different mediator interventions: $\bar{B}_1(t)$, $\bar{B}_2(t)$, $\bar{B}_3(t)$, and $\bar{B}_4(t)$. Each intervention maintains the values of the conditions $W_1, \ldots, W_8$ at the first measurement period $(t = 1)$ at their observed values for each individual. A mediating condition that is set to one at time t remains at one in all subsequent time periods $t+1, \ldots, 16$. Additionally, all of the considered intervention distributions are defined with the censoring indicator set to "never censored".

4.2.3 $\bar{B}_1(T)$: Setting the conditional distributions of all mediators equal to those under always MDD

$\bar{B}_1(T) = \bar{b}_1(16)$ refers to an intervention that iteratively sets the mediator values at each time period t, from the second onwards up to the last, to random draws from conditional counterfactual mediator distributions at t under always MDD. Specifically, the distribution of a mediator is specified to be conditional on baseline covariates, (generated) comorbidities at $t-1$, and on survival at $t-1$ under always MDD and $\bar{B}_1$, i.e., on $D(t-1)^{\bar{z}=\bar{0},\bar{a}=\bar{1},\bar{b}_1} = 0$. For example, at the second time period, $W_s(2) \in \{\boldsymbol{M}(2), \boldsymbol{L}(2)\}$ is set to a random draw from its distribution at $t = 2$ under always MDD, given counterfactual survival at $t = 1$,

$W_s(1) = 0$ and baseline comorbidities $\boldsymbol{W}_{(-s)}(1) = \boldsymbol{w}_{(-s)}(1)$., i.e., from:

$$P(W_s(2)^{\bar{z}=\bar{0},.\bar{a}=\bar{1}} = w_s(2)|\boldsymbol{C} = \boldsymbol{c}, W_s(1) = 0, \boldsymbol{W}_{(-s)}(1) = \boldsymbol{w}_{(-s)}(1), \\ D(1)^{\bar{z}=\bar{0},\bar{a}=\bar{1},\bar{b}_1} = 0). \tag{1}$$

For subsequent time periods, we proceed analogously to generate a counterfactual scenario for the entire follow-up period. In this scenario, the conditional distributions of the mediators at each time period t correspond to those that would be observed under always MDD.

4.2.4 $\bar{B}_2(T)$: Setting the conditional distributions of all mediators equal to those under never MDD

$\bar{B}_2(T) = \bar{b}_2(16)$ refers to an intervention that iteratively sets the mediator values at each time period t, from the second onwards up to the last, to random draws from conditional counterfactual mediator distributions at t under never MDD. Specifically, these distributions are conditional on baseline covariates, (generated) comorbidities at $t-1$, and on survival at $t-1$ under never MDD and $\bar{B}_2$, i.e., on $D(t-1)^{\bar{z}=\bar{0},\bar{a}=\bar{0},\bar{b}_2} = 0$. For example, at the second time period, $W_s(2) \in \{\boldsymbol{M}(2), \boldsymbol{L}(2)\}$ is set to a random draw from its distribution under never MDD at $t = 2$, given counterfactual survival at $t = 1$, $W_s(1) = 0$ and baseline comorbidities $\boldsymbol{W}_{(-s)}(1) = \boldsymbol{w}_{(-s)}(1)$, i.e., from:

$$P(W_s(2)^{\bar{z}=\bar{0},\bar{a}=\bar{1}} = w_s(2)|\boldsymbol{C} = \boldsymbol{c}, W_s(1) = 0, \boldsymbol{W}_{(-s)}(1) = \boldsymbol{w}_{(-s)}(1), \\ D(1)^{\bar{z}=\bar{0},\bar{a}=\bar{0},\bar{b}_2} = 0). \tag{2}$$

Analogously to $\bar{B}_1$, $\bar{B}_2$ generates a counterfactual scenario where the conditional mediator distributions at each time period correspond to those that would be observed in the absence of MDD.

4.2.5 $\bar{B}_3(T)$: Shifting the conditional distributions of the six mediators $M_1, \ldots, M_6$ under always MDD to what would be observed under never MDD

$\bar{B}_3(T) = \bar{B}_3(16)$ refers to an intervention that iteratively shifts the conditional distributions of $M_1, \ldots, M_6$ under always MDD at each time period $t = 2, \ldots, 16$ to those that would be observed if MDD had never occurred, given counterfactual survival at $t-1$ under always MDD and $\bar{B}_3$, i.e., $D(t-1)^{\bar{z}=\bar{0},\bar{a}=\bar{1},\bar{b}_3} = 0$. For example, at the second time period, for all $M_k \in \boldsymbol{M}$, we enforce a random draw $\widetilde{M_k}(2)^{\bar{b}_3}$ upon $M_k(2)$ from the conditional distribution of M_k under never MDD given by:

$$P(M_k(2)^{\bar{z}=\bar{0},\bar{a}=\bar{0}} = m_k(2)|\boldsymbol{C} = \boldsymbol{c}, M_k(1) = 0, \boldsymbol{M}_{(-k)}(1) = \boldsymbol{m}_{(-k)}(1), \\ \boldsymbol{L}(1) = \boldsymbol{l}(1), D(1)^{\bar{z}=\bar{0},\bar{a}=\bar{1},\bar{b}_3} = 0). \tag{3}$$

We allow the interventions on $M_1, \ldots, M_6$ at t to flow into the conditional distributions

of the comorbidities L_1 and L_2 under MDD at $t+1$. This means, for example, that at $t = 3$, L_j, $j \in \{1, 2\}$, is randomly drawn from the conditional distribution

$$P(L_j(3)^{\bar{z}=\bar{0},\bar{a}=\bar{1}} = l_j(3)|\boldsymbol{C} = \boldsymbol{c}, \widetilde{L}_j(2)^{\bar{b}_3} = 0, \widetilde{\boldsymbol{L}}_{(-j)}(2)^{\bar{b}_3} = \boldsymbol{l}_{(-j)}(2),$$
$$\widetilde{\boldsymbol{M}}(2)^{\bar{b}_3} = \boldsymbol{m}(2), D(2)^{\bar{z}=\bar{0},\bar{a}=\bar{1},\bar{b}_3} = 0). \tag{4}$$

4.2.6 $\bar{B}_4(T)$: Shifting the conditional distribution of a single mediator M_k under always MDD to what would be observed under never MDD

$\bar{B}_4(T) = \bar{B}_4(16)$ differs from $\bar{B}_3(T)$ in that it refers to an intervention that iteratively shifts the conditional distribution of a single mediator M_k ($M_k \in \boldsymbol{M}$) at each time period $t = 1, \ldots, 16$ to that expected in the absence of MDD, given $D(t-1)^{\bar{z}=\bar{0},\bar{a}=\bar{1},\bar{b}_4} = 0$. For example, at the second time period, M_k is set to a random draw from the conditional distribution of M_k under never MDD given by:

$$P(M_k(2)^{\bar{z}=\bar{0},\bar{a}=\bar{0}} = m_k(2)|\boldsymbol{C} = \boldsymbol{c}, M_k(1) = 0, \boldsymbol{M}_{(-k)}(1) = \boldsymbol{m}_{(-k)}(1),$$
$$\boldsymbol{L}(1) = \boldsymbol{l}(1), D(1)^{\bar{z}=\bar{0},\bar{a}=\bar{1},\bar{b}_4} = 0). \tag{5}$$

We allow the intervention on the distribution of M_k at t to flow into the conditional distributions of the comorbidities in $\boldsymbol{M}_{(-k)}$ and $\boldsymbol{L}$ under MDD at $t+1$.

4.3 Effect definitions

All effects are defined as contrasts between the expected mortality in two different counterfactual scenarios. In all the considered counterfactual scenarios, individuals who die in time period t remain dead in all subsequent time periods $t+1, \ldots, 16$. The following sections refer to average effects in the entire study population. For the subgroup analyses by sex and age group, the effects are defined to be conditional on sex and age group.

4.3.1 Interventional overall effect of MDD on mortality

We define the average interventional overall effect of MDD on mortality at time t as the contrast between A) the expected mortality at t under an intervention defined by no censoring, the exposure set to always MDD, and the intermediate conditions in $\boldsymbol{M}$ and $\boldsymbol{L}$ set to values specified by $\bar{B}_1$, referred to as the "always MDD" scenario, and B) the expected mortality at t under an intervention defined by no censoring, the exposure set to never MDD, and the distributions of the intermediate conditions in $\boldsymbol{M}$ and $\boldsymbol{L}$ set to those specified by $\bar{B}_2$, referred to as the "never MDD" scenario. On the additive scale, the overall effect can be expressed as a difference given by:

$$Overall(t) \quad = \quad E[D(t)^{\bar{z}=\bar{0},\bar{a}=\bar{1},\bar{b}_1}] - E[D(t)^{\bar{z}=\bar{0},\bar{a}=\bar{0},\bar{b}_2}]. \tag{6}$$

4.3.2 Interventional overall indirect effect through all six mediators

We define the interventional overall indirect effect of MDD on mortality through all six mediators $M_1, \ldots, M_6$ at time t by the contrast between A) the expected mortality at t in the "always MDD" scenario, and C) the expected mortality at t in a counterfactual scenario under always MDD, where the pathways from MDD to death through $M_1, \ldots, M_6$ are eliminated by intervening upon the mediators according to $\bar{B}_3$. On the additive scale, this interventional overall indirect effect through $M_1, \ldots, M_6$ can be expressed as a difference given by:

$$IIE_{\boldsymbol{M}}(t) \quad = \quad E[D(t)^{\bar{z}=\bar{0},\bar{a}=\bar{1},\bar{b}_1}] - E[D(t)^{\bar{z}=\bar{0},\bar{a}=\bar{1},\bar{b}_3}]. \tag{7}$$

In light of recent findings regarding the interpretation of interventional indirect effects [5], we suggest interpreting $IIE_{\boldsymbol{M}}(t)$ as the reduction in the expected mortality in the "always MDD" scenario that would be achieved at time t by an intervention that reduces the conditional risks of the mediators $M_1, \ldots, M_6$ at each time period up to and including t to those that would be observed if MDD had never occurred. The interventional effect of MDD on mortality that would remain after such an intervention is given by $E[D(t)^{\bar{z}=\bar{0},\bar{a}=\bar{1},\bar{b}_3}] - E[D(t)^{\bar{z}=\bar{0},\bar{a}=\bar{0},\bar{b}_2}]$.

4.3.3 Interventional indirect effect through a single mediator

We define the average interventional indirect effect of MDD on mortality through a single mediator M_k, $k \in \{1, \ldots, 6\}$, at time t as the contrast between A) the expected mortality at t in the "always MDD" scenario, and D) the expected mortality at t in a counterfactual scenario under always MDD, where the pathways from MDD to death through M_k are eliminated by intervening upon the mediators according to $\bar{B}_4$. On the additive scale, this interventional indirect effect through M_k can be expressed as a difference given by:

$$IIE_{M_k}(t) \quad = \quad E[D(t)^{\bar{z}=\bar{0},\bar{a}=\bar{1},\bar{b}_1}] - E[D(t)^{\bar{z}=\bar{0},\bar{a}=\bar{1},\bar{b}_4}]. \tag{8}$$

We suggest interpreting $IIE_{M_k}(t)$ as the reduction in the expected mortality in the "always MDD" scenario that would be achieved at time t by an intervention that reduces the conditional risk of mediator M_k at each time period up to and including t to what would be observed if MDD had never occurred. The interventional effect of MDD on mortality that would remain after such an intervention is given by $E[D(t)^{\bar{z}=\bar{0},\bar{a}=\bar{1},\bar{b}_4}] - E[D(t)^{\bar{z}=\bar{0},\bar{a}=\bar{0},\bar{b}_2}]$. According to the structural assumptions of our causal model (Figure 1, main paper), $IIE_{\boldsymbol{M}}(t)$ and $IIE_{M_k}(t)$ are zero at $t = 1$.

4.4 Estimation using a Monte Carlo simulation-based g-computation approach

This section outlines the central steps to estimate the interventional overall effect (equation 6) and interventional indirect effects (equations 7 and 8) using a Monte Carlo simulation-based g-computation approach, adapting the method from Vansteelandt and Daniel [6], who estimated various interventional (in)direct effects in non-longitudinal multiple-mediator settings. G-computation approaches for the estimation of indirect effects in settings with a survival outcome have also been described in Zheng and van der Laan [3] and Lin et al. [1].

We assume that at each time period, the exposure-outcome, mediator-outcome, and exposure-mediator relations are unconfounded given past covariates. Furthermore, we assume positivity of relevant exposure, censoring and mediator values, and consistency [cf. 7, 8]. Identification results for similar contexts involving time-varying mediators and a survival outcome are provided by Zheng and van der Laan [3] for a time-fixed exposure, and by Lin et al. [1] for a time-varying exposure.

Note that the following applies to the estimation algorithm: a disease indicator that is set to one at time period t remains one in all subsequent time periods $t+1, \ldots, 16$. Similarly, individuals estimated to be dead at the end of time period t remain dead in all subsequent time periods $t+1, \ldots, 16$.

<u>Estimation algorithm</u>

1. Specify and fit models for

   (a) the risk of each disease W_s ($W_s \in \boldsymbol{W}, \boldsymbol{W} = (M_1, \ldots, M_6, L_1, L_2)$) for each time period from the second onwards, i.e., for $t = 2, \ldots, 16$ using the observed data. The model for $W_s(t)$ is conditional on:
   1) survival to t, i.e., $D(t-1) = 0$,
   2) MDD and the other comorbidities $\boldsymbol{W}_{(-s)}$ at time $t-1$,
   3) baseline covariates $\boldsymbol{C}$,
   4) no diagnosis of W_s by time period $t-1$, i.e., $W_s(t-1) = 0$.

   (b) death at each time period $t = 1, \ldots, 16$ using the observed data. The outcome model at t is conditional on:
   1) survival to t, i.e., $D(t-1) = 0$,
   2) MDD and $M_1, \ldots, M_6, L_1, L_2$ at time period t,
   3) baseline covariates $\boldsymbol{C}$.

2. Simulate "always MDD" and "never MDD" scenarios to obtain $E[D(t)^{\bar{z}=\bar{0},\bar{a}=\bar{1},\bar{b}_1}]$ and $E[D(t)^{\bar{z}=\bar{0},\bar{a}=\bar{0},\bar{b}_2}]$:

   (a) Set $A(t) = a$ ($a = 1$ for "always MDD" and $a = 0$ for "never MDD") at each time period $t = 1, \ldots, 16$ for each individual.

For each individual at $t = 1$:

(b) Leave the values of $M_1(1), \ldots, M_6(1), L_1(1), L_2(1)$ at their observed status.

(c) Draw the outcome from the outcome model fitted in step 1(b) for $t = 1$, using the updated data generated through steps 2(a)-(b).

Iteratively, for each individual at $t = 2, \ldots, 16$:

(d) For each condition $W_s \in \{M_1, \ldots, M_6, L_1, L_2\}$ draw $W_s(t)$ from the model fitted for W_s in step 1(a) for time period t, using the updated data at $t-1$.

(e) Draw the outcome from the outcome model fitted in step 1(b) for time period t, using the updated data generated through steps 2(a)-(d).

3. Simulate counterfactual scenario under always MDD, setting the conditional risks of $M_1, \ldots, M_6$ equal to those under never MDD, to obtain $E[D(t)^{\bar{z}=\bar{0},\bar{a}=\bar{1},\bar{b}_3}]$:

(a) Set $A(t) = 1$ at each time period $t = 1, \ldots, 16$ for each individual.

For each individual at $t = 1$:

(b) Use data generated through steps 2(b)-(c) where the exposure is set to $A(1) = 1$.

Iteratively, for each individual at $t = 2, \ldots, 16$:

(c) Draw $M_1(t), \ldots, M_6(t)$ from their respective models fitted in step 1(a) for time period t, using the data at $t-1$ generated through steps 2(a)-(d) under the "never MDD" scenario.

(d) Draw $L_1(t)$ and $L_2(t)$ from their respective models fitted in step 1(a) for time period t, using the updated data at $t-1$.

(e) Draw the outcome from the outcome model fitted in step 1(b) for time period t, using the updated data generated through steps 3(a)-(d).

4. Simulate counterfactual scenario under always MDD, setting the conditional risk of a single mediator M_k equal to that under never MDD, to obtain $E[D(t)^{\bar{z}=\bar{0},\bar{a}=\bar{1},\bar{b}_4}]$:

(a) Set $A(t) = 1$ at each time period $t = 1, \ldots, 16$ for each individual.

For each individual at $t = 1$:

(b) Use data generated through steps 2(b)-(c) where the exposure is set to $A(1) = 1$.

<u>Iteratively, for each individual at $t = 2, \ldots, 16$</u>:

(c) Draw $M_k(t)$ from the model fitted for M_k in step 1(a) for time period t, using the data at $t-1$ generated through steps 2(a)-(d) for the “never MDD” scenario.

(d) Draw the conditions in $\boldsymbol{M}_{(-k)}(t)$ and $L_1(t), L_2(t)$ from their respective models fitted in step 1(a) for time period t, using the updated data at $t-1$.

(e) Draw the outcome from the outcome model fitted in step 1(b) for time period t, using the updated data generated through steps 4(a)-(d).

5. Estimate $E[D(t)^{\bar{z}=\bar{0},\bar{a}=\bar{1},\bar{b}_1}]$, $E[D(t)^{\bar{z}=\bar{0},\bar{a}=\bar{0},\bar{b}_2}]$, $E[D(t)^{\bar{z}=\bar{0},\bar{a}=\bar{1},\bar{b}_3}]$ and $E[D(t)^{\bar{z}=\bar{0},\bar{a}=\bar{1},\bar{b}_4}]$ by calculating the proportion of deaths at t in the simulated counterfactuals scenarios. For the subgroup analyses by sex and age group, stratify the expected mortality in the simulated counterfactual scenarios by sex (men and women) and by age group (< 40 and ≥ 40 years of age at enrollment in the study).

6. Obtain estimates for $Overall(t)$, $IIE_{\boldsymbol{M}}(t)$, $IIE_{M_k}(t)$ by subtraction.

We repeated steps 2-6 four times and calculated the mean of the four estimates, following the multiple draws approach outlined in Vansteelandt and Daniel [6] to reduce Monte Carlo error. Confidence intervals were obtained by the nonparametric bootstrap with 1000 bootstrap replicates.

4.5 Results of causal analysis

eTable 2: Estimated interventional overall effect of major depressive disorder (MDD) on mortality, presented on the risk ratio scale and as an absolute difference in percentage points, along with the interventional overall indirect effect through HIV, tuberculosis, diabetes, chronic kidney disease, cardiovascular diseases (CVDs), chronic respiratory diseases, and cancers, presented as a percentage of the overall effect.

Group	Year	Overall effect, risk ratio, (95% CI)	Overall effect, absolute difference in percentage points (95% CI)	Overall indirect effect, percentage of overall effect (95% CI)
Total	1	1.04 (0.94,1.15)	0.03 (-0.05,0.11)	43.0 (-329.4,447.0)
Total	2	1.14 (1.06,1.22)	0.19 (0.09,0.28)	30.0 (18.6,67.2)
Total	3	1.17 (1.11,1.22)	0.33 (0.21,0.43)	37.1 (27.7,58.7)
Total	4	1.20 (1.15,1.25)	0.51 (0.39,0.62)	36.0 (28.0,49.3)
Total	5	1.21 (1.17,1.26)	0.68 (0.54,0.81)	38.0 (30.9,47.7)
Total	6	1.21 (1.17,1.25)	0.83 (0.67,0.97)	41.1 (34.3,50.4)
Total	7	1.22 (1.19,1.26)	1.03 (0.89,1.19)	42.9 (36.7,51.4)
Total	8	1.23 (1.20,1.27)	1.23 (1.06,1.41)	43.4 (38.2,51.0)
Men	1	1.04 (0.93,1.15)	0.03 (-0.06,0.12)	44.7 (-328.6,344.7)
Men	2	1.13 (1.05,1.20)	0.21 (0.08,0.32)	27.3 (14.6,73.6)
Men	3	1.18 (1.10,1.23)	0.40 (0.24,0.52)	32.2 (22.8,54.2)
Men	4	1.23 (1.17,1.28)	0.70 (0.50,0.85)	30.2 (21.6,40.8)
Men	5	1.26 (1.20,1.31)	0.96 (0.77,1.16)	31.6 (23.9,38.2)
Men	6	1.25 (1.20,1.29)	1.15 (0.91,1.32)	36.0 (28.2,42.6)
Men	7	1.27 (1.22,1.31)	1.43 (1.20,1.66)	38.0 (30.8,43.6)
Men	8	1.28 (1.24,1.32)	1.73 (1.47,1.97)	37.3 (31.4,42.8)
Women	1	1.05 (0.94,1.17)	0.03 (-0.03,0.10)	105.3 (-327.8,328.6)
Women	2	1.16 (1.08,1.23)	0.17 (0.09,0.25)	33.7 (19.4,66.4)
Women	3	1.16 (1.10,1.22)	0.26 (0.16,0.36)	44.6 (31.1,69.3)
Women	4	1.16 (1.10,1.21)	0.34 (0.22,0.44)	47.6 (35.7,75.4)
Women	5	1.15 (1.10,1.20)	0.41 (0.29,0.53)	52.4 (42.3,80.1)
Women	6	1.16 (1.12,1.21)	0.53 (0.41,0.67)	51.5 (43.8,72.7)
Women	7	1.17 (1.13,1.21)	0.65 (0.53,0.82)	52.9 (46.1,71.0)
Women	8	1.17 (1.14,1.21)	0.76 (0.63,0.94)	56.2 (49.1,73.3)
$<$40 years	1	0.99 (0.85,1.14)	0.00 (-0.04,0.04)	88.5 (-598.9,718.4)
$<$40 years	2	1.11 (1.00,1.21)	0.05 (0.00,0.10)	37.6 (15.6,340.2)
$<$40 years	3	1.14 (1.04,1.23)	0.09 (0.03,0.15)	55.2 (33.7,160.2)
$<$40 years	4	1.19 (1.11,1.27)	0.16 (0.10,0.23)	49.1 (35.6,84.7)
$<$40 years	5	1.21 (1.14,1.29)	0.22 (0.15,0.30)	51.3 (40.7,76.9)
$<$40 years	6	1.22 (1.16,1.29)	0.27 (0.20,0.36)	54.1 (43.5,75.2)
$<$40 years	7	1.23 (1.18,1.31)	0.34 (0.27,0.45)	55.7 (46.6,73.9)
$<$40 years	8	1.26 (1.21,1.32)	0.43 (0.36,0.54)	54.9 (47.2,69.5)
$\geq$40 years	1	1.06 (0.95,1.17)	0.07 (-0.06,0.21)	33.9 (-226.4,338.7)
$\geq$40 years	2	1.15 (1.07,1.22)	0.36 (0.18,0.53)	28.7 (16.2,56.2)
$\geq$40 years	3	1.18 (1.11,1.22)	0.63 (0.41,0.80)	33.9 (24.0,52.2)
$\geq$40 years	4	1.20 (1.15,1.24)	0.96 (0.71,1.13)	33.3 (25.0,45.2)
$\geq$40 years	5	1.21 (1.17,1.25)	1.25 (1.00,1.48)	35.1 (27.4,43.1)
$\geq$40 years	6	1.21 (1.17 1.25)	1.52 (1.25,1.76)	38.2 (31.1,46.4)
$\geq$40 years	7	1.22 (1.19,1.26)	1.88 (1.61,2.16)	40.0 (33.7,47.2)
$\geq$40 years	8	1.23 (1.19,1.26)	2.23 (1.92,2.52)	40.6 (34.9,47.8)

eTable 3: Estimated interventional indirect effects (IIEs) of major depressive disorder (MDD) on mortality through single mediators HIV, tuberculosis, diabetes, chronic kidney disease, cardiovascular diseases (CVDs), chronic respiratory diseases, and cancers, presented as percentages of the interventional overall effect, with 95% confidence intervals.

Group	Year	IIE HIV	IIE Tuberculosis	IIE Diabetes & Chronic kidney disease	IIE CVDs	IIE Chronic respiratory diseases	IIE Cancers
Total	1	18.8 (-172.4,186.4)	16.4 (-150.1,211.5)	2.3 (-144.3,141.6)	28.1 (-206.7,276.7)	25.8 (-145.5,179.8)	7.3 (-180.2,125.4)
Total	2	0.9 (-6.5,13.6)	7.5 (-5.1,17.5)	1.9 (-8.6,11.5)	9.0 (3.1,29.7)	11.4 (-1.4,19.8)	7.4 (-3.4,18.5)
Total	3	2.9 (-2.2,10.7)	5.0 (-1.1,13.4)	2.8 (-4.7,9.1)	10.4 (7.0,23.3)	11.8 (2.5,17.5)	6.0 (0.6,16.1)
Total	4	3.1 (-1.5,7.9)	7.0 (0.2,10.4)	5.8 (-1.4,8.5)	11.9 (8.3,19.4)	10.9 (4.0,14.3)	6.0 (1.8,12.8)
Total	5	3.4 (-0.9,6.9)	7.3 (0.8,9.8)	5.5 (-0.1,8.2)	14.8 (9.6,19.4)	9.9 (4.6,12.5)	7.8 (3.2,12.4)
Total	6	3.2 (-0.2,6.7)	5.2 (1.5,9.2)	5.1 (1.2,8.7)	17.5 (12.4,21.3)	9.4 (5.5,12.4)	6.4 (3.8,11.8)
Total	7	3.0 (0.1,6.0)	4.9 (2.2,8.7)	5.9 (2.8,9.3)	16.8 (13.4,21.4)	9.6 (6.3,12.5)	7.1 (4.4,11.4)
Total	8	2.7 (0.5,5.7)	4.3 (2.3,8.1)	5.8 (3.3,9.4)	17.8 (14.5,22.1)	8.6 (6.4,11.9)	7.5 (5.0,11.1)
Men	1	16.3 (-159.9,251.1)	11.2 (-169.0,246.6)	-2.9 (-142.3,272.6)	18.1 (-274.5,331.0)	4.0 (-193.9,244.6)	5.8 (-167.0,301.5)
Men	2	-0.9 (-11.1,21.4)	7.5 (-8.5,23.1)	1.1 (-14.6,17.5)	2.8 (0.9,35.0)	7.1 (-5.2,31.2)	2.8 (-17.2,15.9)
Men	3	2.9 (-4.0,12.5)	4.9 (-2.9,15.8)	3.4 (-5.7,10.8)	7.4 (5.6,24.4)	8.5 (1.2,19.5)	1.9 (-7.8,11.8)
Men	4	1.3 (-2.3,8.5)	5.0 (-0.2,10.7)	5.2 (-2.4,8.1)	7.9 (6.1,17.5)	7.7 (2.5,14.1)	1.3 (-3.7,9.2)
Men	5	3.0 (-1.1,7.1)	4.3 (0.5,9.2)	5.3 (-1.2,7.1)	11.9 (7.7,16.7)	7.8 (3.5,11.5)	3.7 (-1.6,8.2)
Men	6	3.3 (-0.5,7.1)	3.5 (1.1,9.0)	5.2 (-0.2,7.9)	14.8 (10.2,19.0)	8.4 (4.3,11.9)	3.5 (-0.8,8.5)
Men	7	2.7 (-0.2,6.6)	3.8 (2.0,8.9)	5.7 (1.6,8.4)	14.2 (11.0,18.9)	9.1 (5.2,12.1)	4.3 (0.2,8.3)
Men	8	2.0 (0.1,6.3)	3.4 (2.1,8.2)	5.1 (2.1,8.3)	14.4 (11.8,19.1)	7.1 (5.0,11.4)	4.2 (1.1,8.0)
Women	1	37.5 (-144.1,134.8)	33.5 (-120.4,130.8)	0.3 (-124.0,114.8)	45.3 (-164.1,158.1)	66.8 (-130.9,147.4)	0.1 (-150.7,203.7)
Women	2	3.6 (-13.1,14.1)	7.0 (-10.4,16.4)	1.9 (-10.8,13.7)	16.5 (-0.6,27.7)	15.9 (-6.6,20.3)	12.1 (-0.5,31.4)
Women	3	3.3 (-7.8,12.4)	4.7 (-5.9,15.8)	1.4 (-7.5,12.7)	15.0 (4.9,26.2)	16.5 (-0.6,19.4)	11.3 (3.9,26.9)
Women	4	7.1 (-5.0,12.0)	10.7 (-4.1,14.6)	6.5 (-4.0,13.4)	20.1 (8.2,27.2)	17.3 (0.6,20.2)	15.2 (6.5,27.2)
Women	5	4.6 (-4.7,11.3)	14.1 (-3.2,14.7)	5.3 (-1.3,14.6)	21.8 (11.5,30.3)	14.6 (3.0,19.2)	17.1 (9.3,28.7)
Women	6	3.1 (-3.1,8.9)	8.9 (-1.4,12.1)	4.8 (0.6,13.1)	23.3 (14.0,29.2)	11.4 (3.8,17.5)	12.6 (9.1,24.2)
Women	7	3.5 (-2.7,8.0)	6.9 (-0.3,11.4)	6.0 (2.5,14.1)	22.4 (15.9,29.6)	10.6 (4.6,16.9)	13.0 (9.1,22.2)
Women	8	4.0 (-1.8,7.9)	6.3 (0.3,10.7)	7.2 (3.6,14.7)	25.2 (17.6,30.6)	11.9 (5.6,17.0)	14.6 (9.7,21.9)
<40 years	1	-544.3 (-485.4,455.7)	658.3 (-562.0,327.9)	1067.4 (-447.4,370.1)	301.7 (-436.4,484.7)	47.6 (-435.8,345.2)	-372.6 (-366.4,397.8)
<40 years	2	8.3 (-47.8,109.6)	14.6 (-47.7,77.6)	2.4 (-48.8,106.9)	19.9 (-39.5,76.2)	4.5 (-54.4,67)	10.0 (-79.6,56.3)
<40 years	3	18.5 (-3.6,46.8)	19.4 (-8.0,48.7)	14.0 (-8.9,46.4)	16.7 (-5.7,41.3)	10.6 (-12.0,35.2)	6.8 (-12.9,36.3)
<40 years	4	19.2 (-1.5,24.6)	19.0 (-1.0,24.8)	16.6 (0.5,28.2)	16.7 (0.9,24.2)	9.9 (-5.0,19.7)	6.6 (-4.5,19.1)
<40 years	5	17.3 (2.7,21.9)	17.0 (0.8,21.7)	14.6 (4.8,25.6)	14.8 (2.9,22.3)	6.1 (-2.0,16.7)	6.1 (-2.2,17.7)
<40 years	6	16.8 (3.6,19.9)	15.7 (1.6,20.4)	15.9 (6.7,25.4)	18.6 (6.5,23.7)	4.2 (-1.3,14.9)	5.9 (0.2,16.2)
<40 years	7	15.9 (3.6,18.0)	15.1 (2.7,18.7)	16.2 (9.5,25.1)	19.3 (9.2,23.6)	5.6 (1.1,14.9)	6.7 (1.6,15.8)
<40 years	8	14.0 (4.0,16.1)	13.8 (3.3,16.4)	16.1 (10.0,23.9)	19.7 (10.5,23.3)	6.2 (1.9,13.4)	7.5 (2.6,15.2)
≥40 years	1	15.9 (-154.0,84.6)	8.4 (-99.5,112.4)	-0.4 (-139.7,85.1)	23.7 (-162.2,203.2)	21.5 (-124.8,129.6)	4.2 (-108.3,88.4)
≥40 years	2	-1.5 (-9.4,11.4)	5.3 (-7.6,14.0)	-0.3 (-11.0,9.5)	7.1 (3.1,28.2)	11.4 (-1.3,20.4)	6.5 (-2.8,18.8)
≥40 years	3	-0.2 (-4.9,8.5)	2.1 (-2.4,11.4)	0.2 (-6.6,7.3)	9.2 (6.7,22.2)	11.8 (3.2,17.3)	5.7 (0.3,15.2)
≥40 years	4	-0.3 (-3.5,6.1)	4.5 (-0.7,9.0)	3.6 (-3.7,6.4)	10.9 (8.1,19.6)	11.2 (4.0,15.0)	6.0 (1.7,13.0)
≥40 years	5	0.4 (-2.6,4.8)	5.3 (-0.8,8.3)	3.5 (-2.6,6.2)	14.8 (10.0,19.4)	10.7 (4.5,13.2)	8.2 (3.0,12.7)
≥40 years	6	0.2 (-2.1,4.6)	2.9 (-0.1,8.2)	2.7 (-1.6,6.3)	17.3 (12.6,21.5)	10.5 (5.6,13.4)	6.6 (3.7,12.0)
≥40 years	7	0.1 (-1.4,4.2)	2.5 (0.9,7.7)	3.5 (0.0,6.9)	16.3 (13.4,21.6)	10.4 (6.1,13.2)	7.2 (4.3,11.5)
≥40 years	8	-0.1 (-1.2,4.1)	2.1 (1.3,7.2)	3.3 (0.6,7.3)	17.4 (14.9,22.2)	9.2 (6.3,12.7)	7.5 (4.7,11.4)

5 Results of sensitivity analysis

This section shows the results of a sensitivity analysis, which was based on a random 50% sample of the study population. In the sensitivity analysis, we relaxed the assumption that MDD at time period t could only have affected the risk of being diagnosed with another disease in the subsequent period. Instead, we assumed that MDD diagnosed at time period t ($t \geq 2$) might be the cause of another disease diagnosed within the same time period t. As in the main analysis, comorbidities of MDD diagnosed within the first time period ($t = 1$) were modeled as baseline conditions that were not intervened on.

eFigure 2: Panel A and B of this figure show the estimated interventional overall effects at 1 to 8 years of follow-up defined as the difference in all-cause mortality risk between two counterfactual scenarios: everyone being exposed to major depressive disorder (MDD) ("always MDD" scenario) versus no-one being exposed to MDD throughout follow-up ("never MDD" scenario). Panel A presents the overall effect as an absolute difference in mortality as percentage points, while Panel B represents the relative difference in mortality expressed as risk ratio. Panel C of this figure shows the interventional overall indirect effects at 3 to 8 years of follow-up, defined as the percentage reductions in the interventional overall effect achieved by setting the risks of HIV, tuberculosis, diabetes & chronic kidney disease, cardiovascular diseases, chronic respiratory diseases, and cancers under always MDD equal to those that would be observed in the absence of MDD. The error bars indicate the 95% confidence intervals.

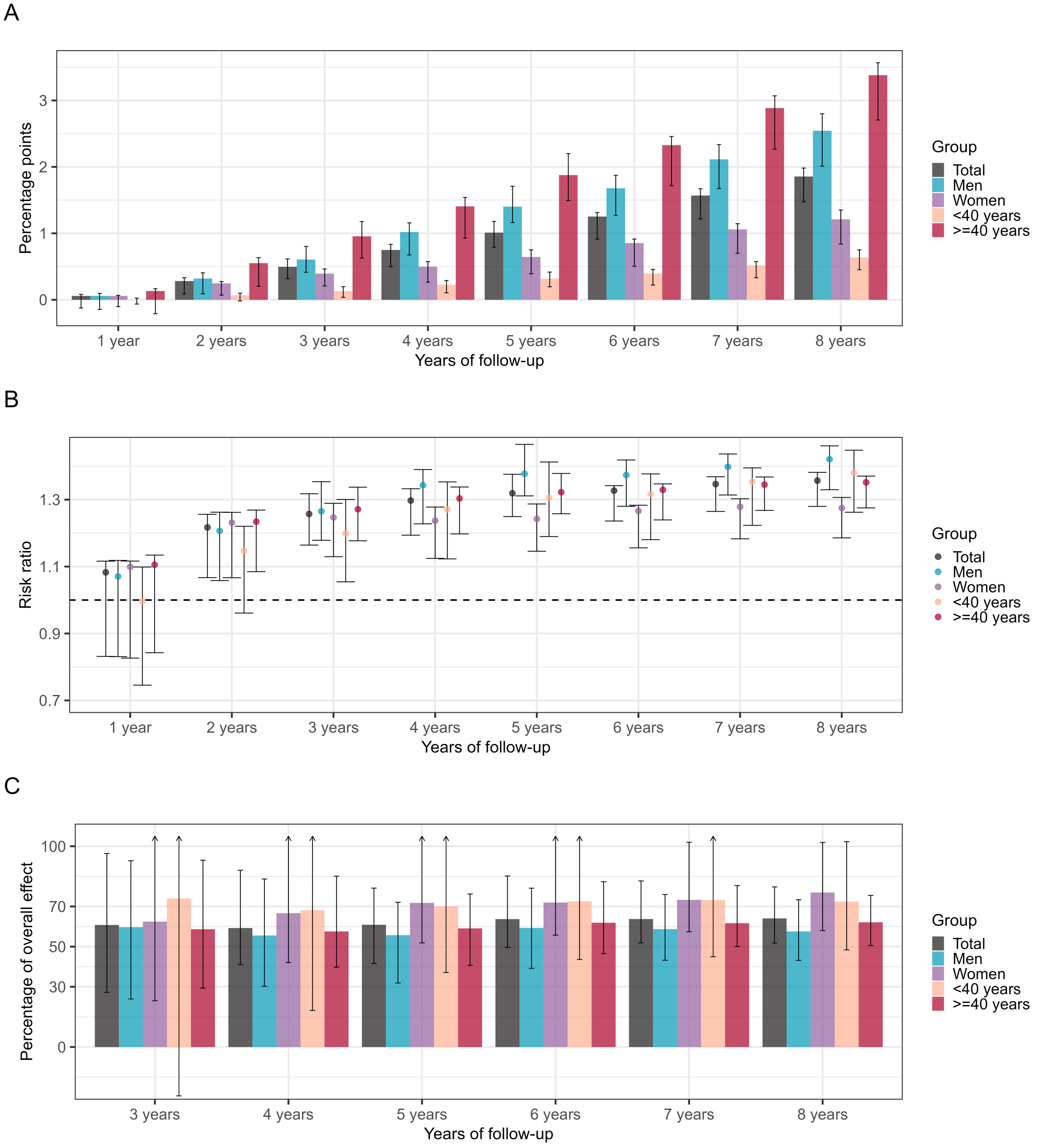

eFigure 3: This figure shows the estimated interventional indirect effects of major depressive disorder (MDD) on mortality through six physical diseases at 3 to 8 years of follow-up. The interventional indirect effect through a physical disease in a given year is defined as the reduction in the mortality risk under always MDD for that year, achieved by setting the risk of that disease over the entire follow-up period to the level that would be observed in the absence of MDD. The coloured bars represent the indirect effects as percentages of the interventional overall effect of MDD on mortality. Panel A presents results for the entire study population, Panel B is stratified by sex, and Panel C by age groups

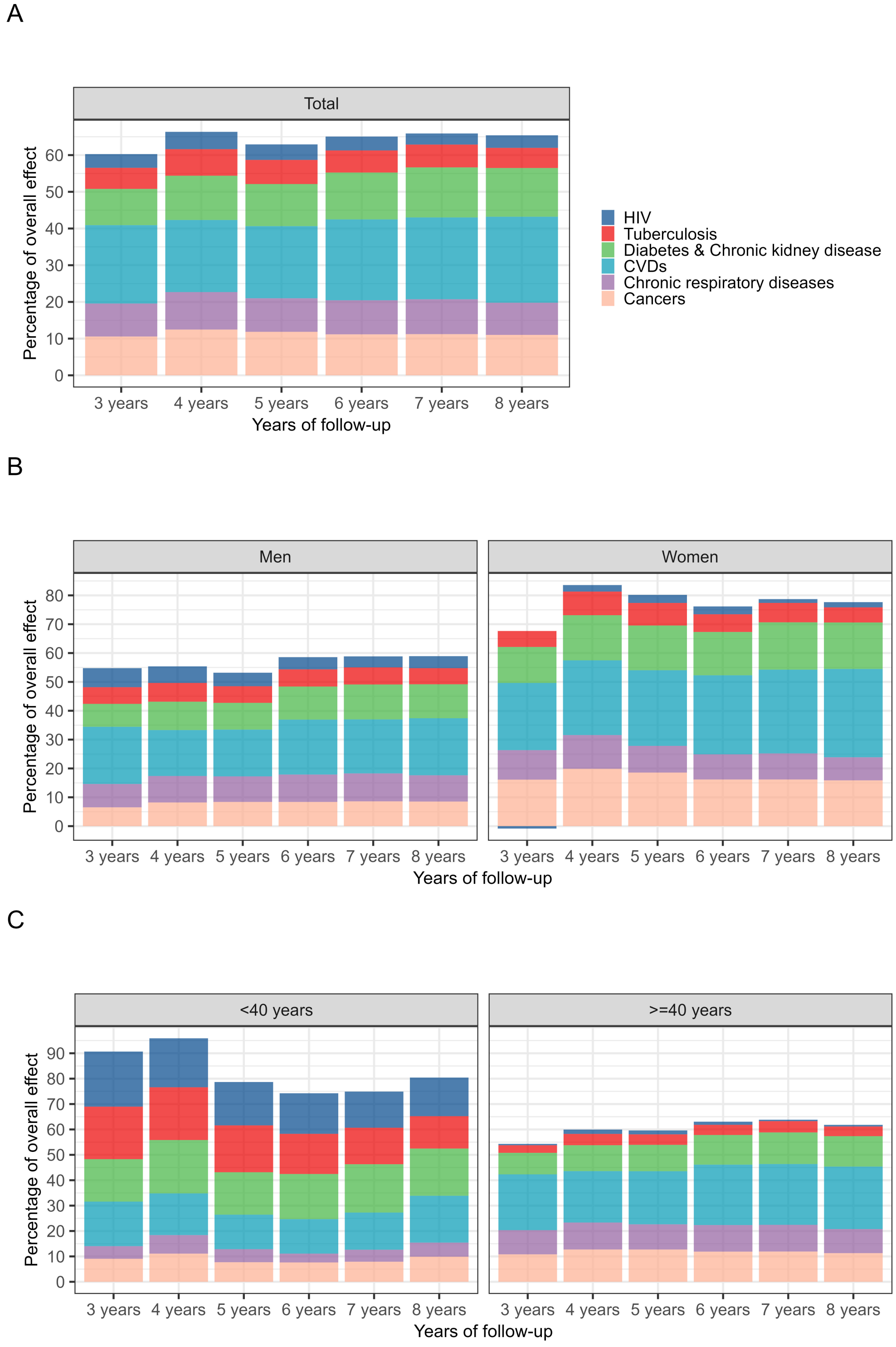

eFigure 4: This figure shows the estimated interventional indirect effects of major depressive disorder (MDD) on mortality through six physical diseases at year 3 and year 8 of follow-up with 95% confidence intervals. The interventional indirect effect through a physical disease in a given year is defined as the reduction in the mortality risk under always MDD for that year, achieved by setting the risk of that disease over the entire follow-up period to the level that would be observed in the absence of MDD. The coloured bars represent the indirect effects as percentages of the interventional overall effect of MDD on mortality. Panel A presents results for the entire study population, Panel B is stratified by sex, and Panel C by age groups.

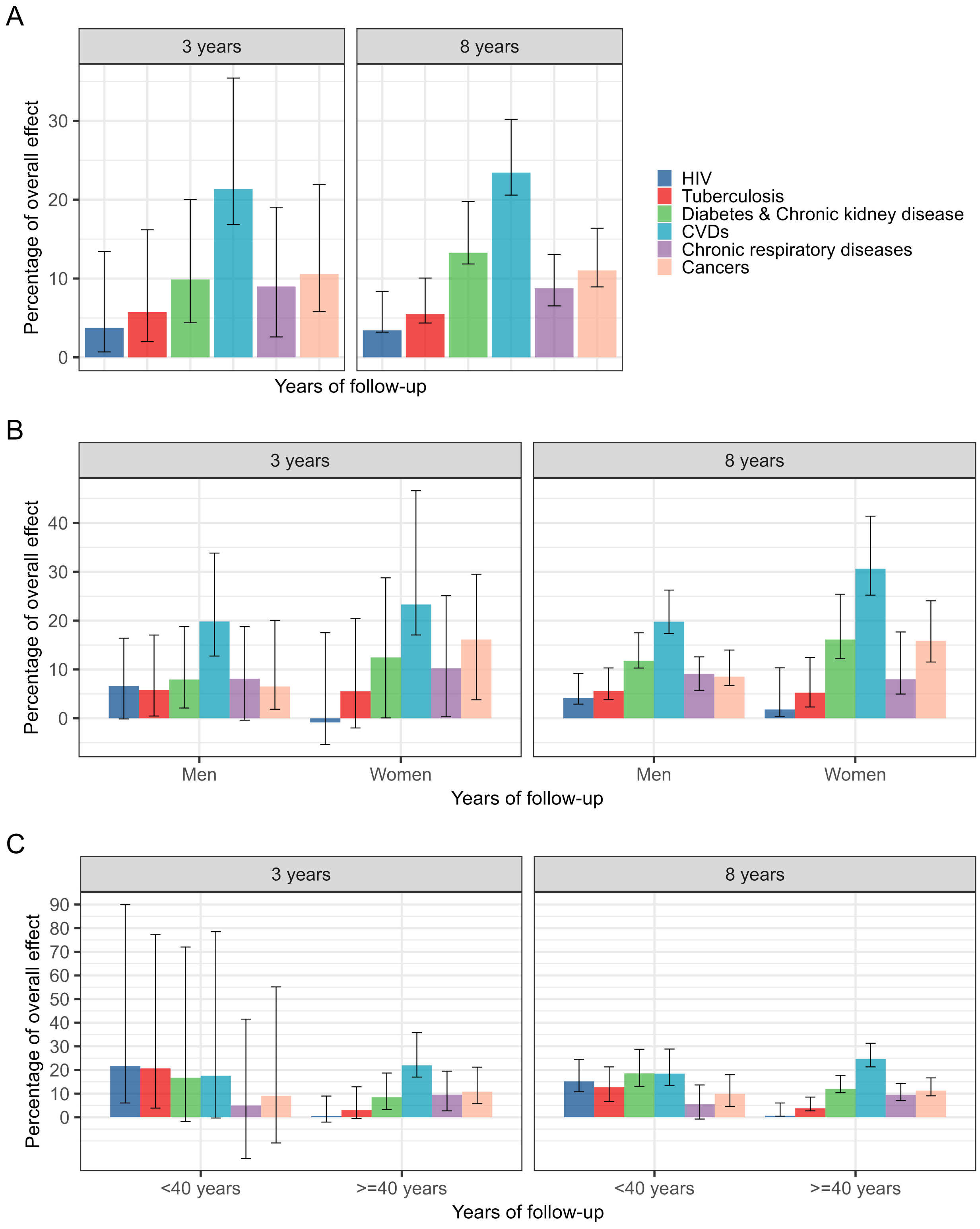

References

[1] Sheng-Hsuan Lin, Jessica G. Young, Roger Logan, and Tyler J. VanderWeele. Mediation analysis for a survival outcome with time-varying exposures, mediators, and confounders. *Statistics in Medicine*, 36 (26):4153–4166, 2017.

[2] Tyler J. VanderWeele and Eric J. Tchetgen Tchetgen. Mediation analysis with time varying exposures and mediators. *Journal of the Royal Statistical Society: Series B (Statistical Methodology)*, 79(3):917–938, 2017.

[3] Wenjing Zheng and Mark J. van der Laan. Causal mediation in a survival setting with time-dependent mediators. Working Paper 295, U.C. Berkeley Division of Biostatistics Working Paper Series, 2012.

[4] Margarita Moreno-Betancur, Paul Moran, Denise Becker, George C. Patton, and John B. Carlin. Mediation effects that emulate a target randomised trial: Simulation-based evaluation of ill-defined interventions on multiple mediators. *Statistical Methods in Medical Research*, 30(6):1395–1412, 2021.

[5] Caleb H Miles. On the causal interpretation of randomised interventional indirect effects. *Journal of the Royal Statistical Society Series B: Statistical Methodology*, 85(4):1154–1172, 2023.

[6] Stijn Vansteelandt and R. M. Daniel. Interventional effects for mediation analysis with multiple mediators. *Epidemiology*, 28(2):258–265, 2017.

[7] Stephen R. Cole and Constantine E. Frangakis. The Consistency Statement in Causal Inference: A Definition or an Assumption? *Epidemiology*, 20(1):3–5, 2009.

[8] Trang Quynh Nguyen, Ian Schmid, Elizabeth L. Ogburn, and Elizabeth A. Stuart. Clarifying causal mediation analysis: Effect identification via three assumptions and five potential outcomes. *Journal of Causal Inference*, 10(1):246–279, 2022.
